# Solvent-Induced Pathway Complexity of Supramolecular Polymerization Unveiled Using the Hansen Solubility Parameters

**DOI:** 10.1021/jacs.3c05547

**Published:** 2023-08-02

**Authors:** Joost J. B. van der Tol, Ghislaine Vantomme, E. W. Meijer

**Affiliations:** † Institute for Complex Molecular Systems and Laboratory of Macromolecular and Organic Chemistry, Eindhoven University of Technology, P.O. Box 513, Eindhoven 5600 MB, The Netherlands; ‡ School of Chemistry and RNA Institute The University of New South Wales, Sydney, New South Wales 2052, Australia

## Abstract

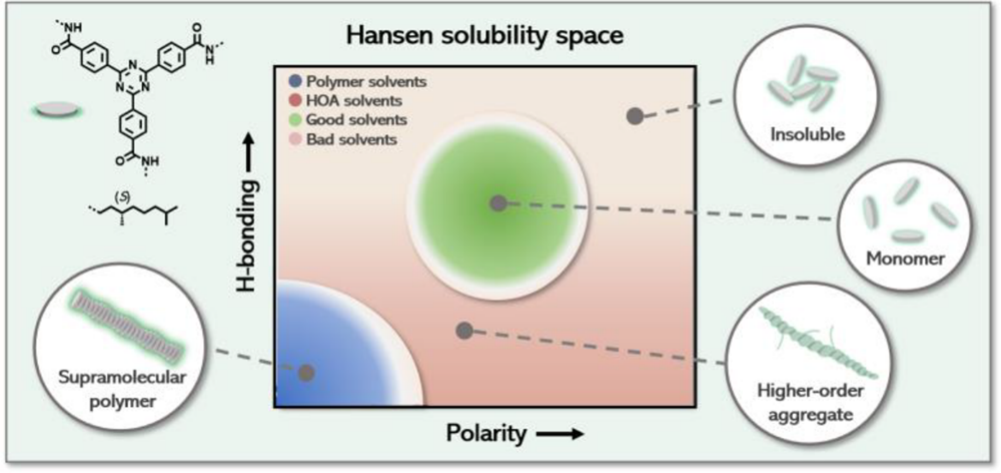

Supramolecular building blocks assembling into helical aggregates are ubiquitous in the current literature, yet the role of solvents in these supramolecular polymerizations often remains elusive. Here, we present a systematic study that quantifies solvent–supramolecular polymer compatibility using the Hansen solubility parameters (δ_D_, δ_H_, and δ_P_). We first studied the solubility space of the supramolecular building block triazine-1,3,5-tribenzenecarboxamide ***S*-T**. Due to its amphiphilic nature, a dual-sphere model based on 58 solvents was applied describing the solubility space of the monomeric state (green sphere) and supramolecular polymer state (blue sphere). To our surprise, further in-depth spectroscopic and morphological studies unveiled a distinct solubility region in-between the two spheres giving rise to the formation of higher-order aggregated structures. This phenomenon occurs due to subtle differences in polarity between the solvent and the side chains and highlights the solvent-induced pathway complexity of supramolecular polymerizations. Subsequent variations in concentration and temperature led to the expansion and contraction of both solubility spheres providing two additional features to tune the monomer and supramolecular polymer solubility. Finally, we applied our dual-sphere model on structurally disparate monomers, such as Zn-porphyrin (***S*-P**) and triphenylamine (***S*-A**), demonstrating the generality of the model and the importance of the supramolecular monomer design in connection with the solvent used. This work unravels the solvent-induced pathway complexity of discotic supramolecular building blocks using a parametrized approach in which interactions between the solvent and solute play a crucial role.

## Introduction

Supramolecular polymers have emerged at the end of the last century and attracted a lot of interest^1,2^ due to their potential applications in various fields ranging from biomaterials to optoelectronics.^3−8^ In particular, the cooperative supramolecular systems have inspired many researchers to study their assembly, which closely resembles processes found in nature, i.e., in microtubules and actin filaments. Besides their intricate kinetics and dynamics, supramolecular polymers can assemble into various morphologies with distinct properties, depending not only on the monomers’ structure but also on the pathway selected during the sample preparation.^9^ More recently, functional supramolecular building blocks have been synthesized comprising exciting properties such as conductivity,^10^ exciton migration,^11^ and bulk photovoltaic effect,^12^ showing their great potential to bring new functions into devices.

However, to process functional supramolecular materials, highly concentrated solutions are required and often in combination with co-monomers, polymers, or additives, which limit the solubility prediction of both the monomers and supramolecular polymers. Additionally, supramolecular systems are often restricted to studies in dilute conditions and apolar solvents^13,14^ due to their high sensitivity to impurities^15^ and susceptibility for kinetic traps and complex assembly pathways.^16−18^ Therefore, fundamental knowledge on the manyfold of interactions within such a system, i.e., solvent–solute, solute–solute, and solvent–solvent,^19,20^ is crucial for the development of multicomponent functional materials^1^ and understanding the pathway complexity^21,22^ that comes along with this challenge. These interactions become particularly important in the case of amphiphilic molecules, which constitute the majority of supramolecular building blocks, because of the distinct affinities of organic solvents toward the side chains and assembling core.^23^ Depending on the assembly conditions, these amphiphilic molecules are either reported as well-defined individual fibers or higher-order aggregates while it remains unclear how these cases are related.

A first foundation for the quantitative evaluation of solute–solvent, or rather polymer–solvent interactions, was established by Hildebrand known as the Hildebrand parameters.^24^ Later, many other solvent description models were developed such as Reichardt’s dye,^25^ the Kamlet–Taft parameters^26−28^ and the Hansen solubility parameters.^29^ The latter has been presented by Charles Hansen and defines the solubility parameter by the cohesive energy density holding the liquid together. These forces describe the chemical nature of solvent molecules by three parameters: dispersion (London) forces (δ_D_), polar forces (δ_P_), and hydrogen bonding (δ_H_) and have proven to be sufficient to describe the solubility of polymers.^30^ As a result, the Hansen solubility parameters (HSPs) have been widely applied in the polymer and coating industry^31−33^ and have recently been used to study the solubility of drugs,^34−36^ inorganic dispersions,^37^ and molecular gels.^38−41^ Recent work from the groups of Rogers^42^ and Bouteiller^43^ shows that by using an HSP approach, the formation of organogels from low molecular weight gelators could be successfully rationalized and predicted. However, for one-dimensional supramolecular assemblies, the choice of solvent remains an educated guess from a “trial and error” method. Hence, a systematic approach providing a fundamental understanding about these supramolecular systems has become indispensable.

**Figure 1:**
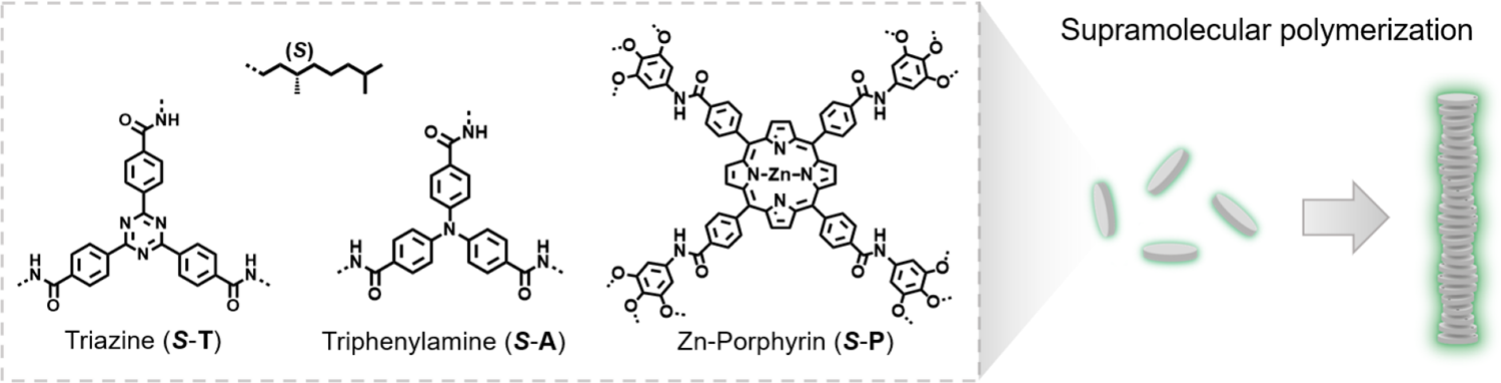
Molecular structures of supramolecular building blocks used in this study to unravel the solubility and pathway complexity of supramolecular polymers.

Here, we present a systematic study using a parametrized method to rationalize the effect of solvents on supramolecular building blocks (Figure 1). Because of the similitudes between covalent and supramolecular polymers,^44^ we were incited to explore if the HSP approach, so successful for covalent polymers, can also help unravel the solubility and pathway complexity of supramolecular polymers. For this purpose, we investigate the solubility of triazine-1,3,5-tribenzenecarboxamide decorated with chiral alkyl chains (***S*-T**)^45^ in 58 different solvents with known HSPs using a combination of qualitative solubility evaluation, UV–vis, circular dichroism (CD) spectroscopy, and atomic force microscopy (AFM). The “de los Rios” algorithm^46^ was subsequently used to determine the unknown HSPs of ***S*-T**, including the action radius (*R*_0_). In the next step, general trends were derived from the corresponding Hansen solubility space of ***S*-T** followed by probing the effect of concentration and temperature. Finally, we determined the Hansen solubility spaces for Zn-porphyrin (***S*-P**) and triphenylamine (***S*-A**) to demonstrate the general applicability of the HSP model toward discotic supramolecular building blocks.

## Results and Discussion

### Preliminary Solvent Studies

**Figure 2:**
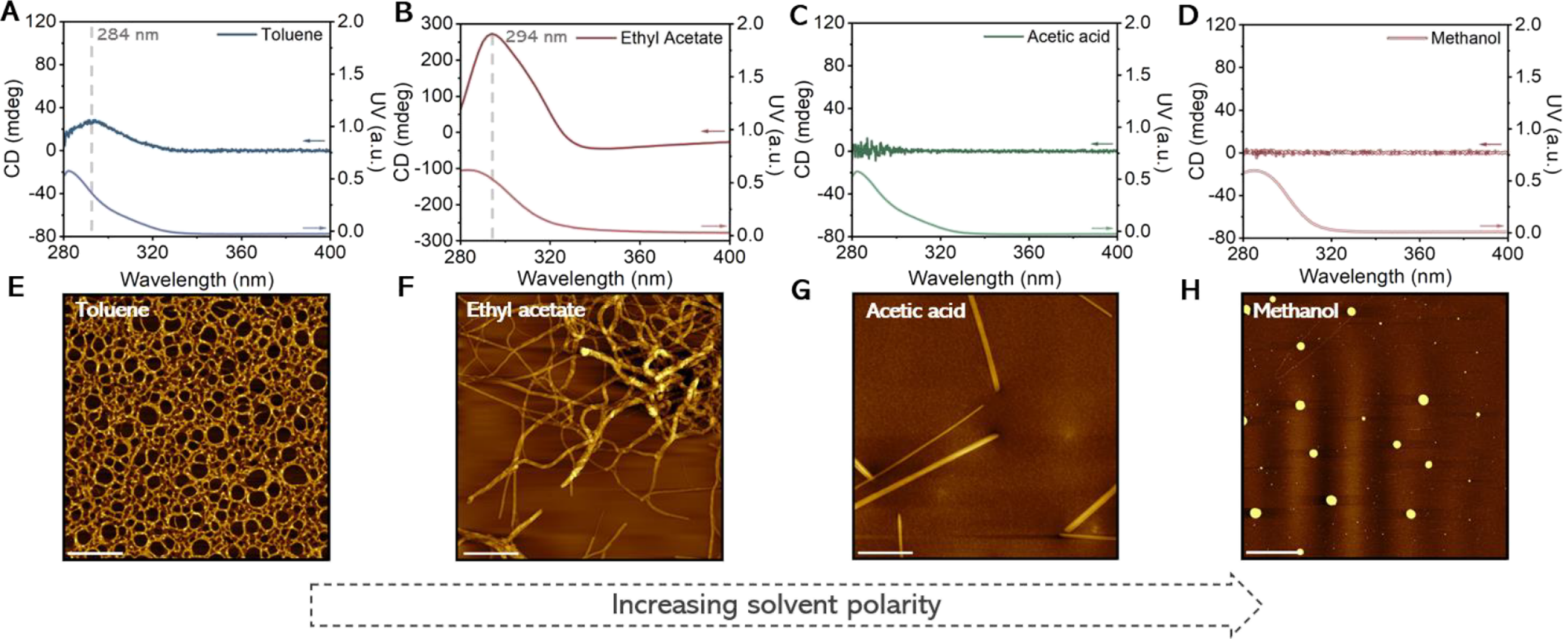
CD and UV spectra of 100 μM solutions of ***S*-T** in (A) toluene, (B) ethyl acetate, (C) acetic acid, and (D) methanol. 10 × 10 μm AFM height images of dropcasted samples from (E) toluene, (F) ethyl acetate, (G) acetic acid, and (H) methanol displaying the solvent-dependent morphology of ***S*-T**. The inset scalebar represents 2 μm.

To investigate the role of solvents on discotic supramolecular building blocks, we initially performed a preliminary solubility study at room temperature employing four common solvents (toluene, ethyl acetate, acetic acid, and methanol) with varying polarity (Figure 2). The chiral supramolecular monomer ***S*-T**(45) was chosen as the model compound (Figure 1) due to the high stability of the assembled state in apolar solvents and insensitivity to kinetic traps. Samples were prepared by heating 100 μM solutions of ***S*-T** in each solvent to the corresponding boiling point for 10 s, followed by slow ambient cooling to room temperature and equilibration for 30 min. First, the ***S*-T** solutions were visually inspected in the presence or absence of precipitates categorizing the solvents as bad or good solvents, respectively. To further distinguish between the molecularly dissolved and supramolecular polymer state in case of ***S*-T** solubilization, we conducted CD and UV spectroscopy supported by AFM characterization on dropcasted samples prepared from 100 μM solutions of ***S*-T**.

**Figure 3:**
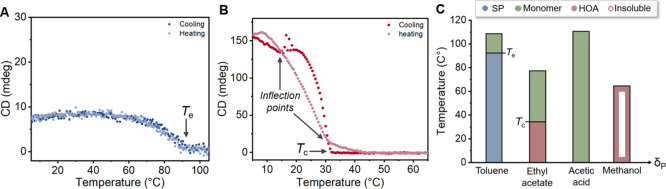
Controlled heating and cooling runs (1 °C min^–1^) of 50 μM solutions of ***S*-T** in (A) toluene and (B) ethyl acetate. (C) Morphological states of ***S*-T** as a function of temperature and solvent polarity in which SP and HOA represent the supramolecular polymer and higher-order aggregate state, respectively. The cooling runs of ***S*-T** in acetic acid and methanol are shown in Figures S1C and S1D. The CD signal was followed at 284 nm for toluene, acetic acid, and methanol and at 296 nm for ethyl acetate. The *T*_e_ and *T*_c_ represent the elongation temperature of supramolecular polymerization and the critical assembly temperature at which higher order aggregates start to form, respectively.

Toluene is a low polarity solvent (δ_P_ = 1.4) commonly applied to induce the assembly of supramolecular building blocks, which was confirmed for ***S*-T** by the absence of precipitation and presence of a CD signal (Figure 2A). Subsequent AFM studies supported this claim by displaying a network-like structure typically originating from a solution containing single supramolecular polymer chains (Figures 2E and S9).^45^ Temperature-dependent CD spectroscopy on a 50 μM solution of ***S*-T** presented a corresponding elongation temperature (*T*_e_) at 94 °C (Figure 3A) characteristic for a cooperative supramolecular polymerization. For ethyl acetate (δ_P_ = 5.3), a long fibrous precipitate was observed after a few minutes at room temperature. Unexpectedly, the initially clear solution displayed a Cotton effect differing in intensity, CD maximum, and shape (as visible by the appearance of a shoulder centered at 310 nm) compared to the solution of ***S*-T** in toluene (Figure 2B). AFM topology images revealed the formation of large assembled fibers of ***S*-T** (Figures 2F, S2, and S10), known as “super helices”^16^ or “higher-order aggregates” (HOAs), which showed a critical assembly temperature *T*_c_ at 32 °C upon cooling (Figure 3B). The subsequent heating run, however, showed a large hysteresis effect, suggesting that the formation of the HOAs is kinetically controlled. Moreover, an inflection point at the *T*_c_ was observed followed by a similar decrease in CD signal as presented for the depolymerization of a single chain polymer of ***S*-T** in toluene (Figure 3A). This observation hints toward the hierarchical assembly of ***S*-T** polymers into HOAs. By further increasing the polarity of the solvent using acetic acid (δ_P_ = 8), neither in a precipitate nor a CD signal was observed implying the solvation of the monomer ***S*-T** (Figure 2C). Subsequent dropcasting of ***S*-T** from acetic acid resulted in assemblies (Figures 2G and S11), which are generally observed upon concentrating a molecularly dissolved solution. In the highest polarity solvent, methanol (δ_P_ = 12.3), the absence of a CD signal (Figure 2D) and the observation of globular aggregates on the surface (Figures 2H and S12) were in line with the presence of precipitates of ***S*-T** in solution. The morphology of ***S*-T** in the four solvents as function of temperature is presented in Figure 3C, which illustrates the four distinct morphologies at room temperature.

### Applying the HSPs

**Figure 4:**
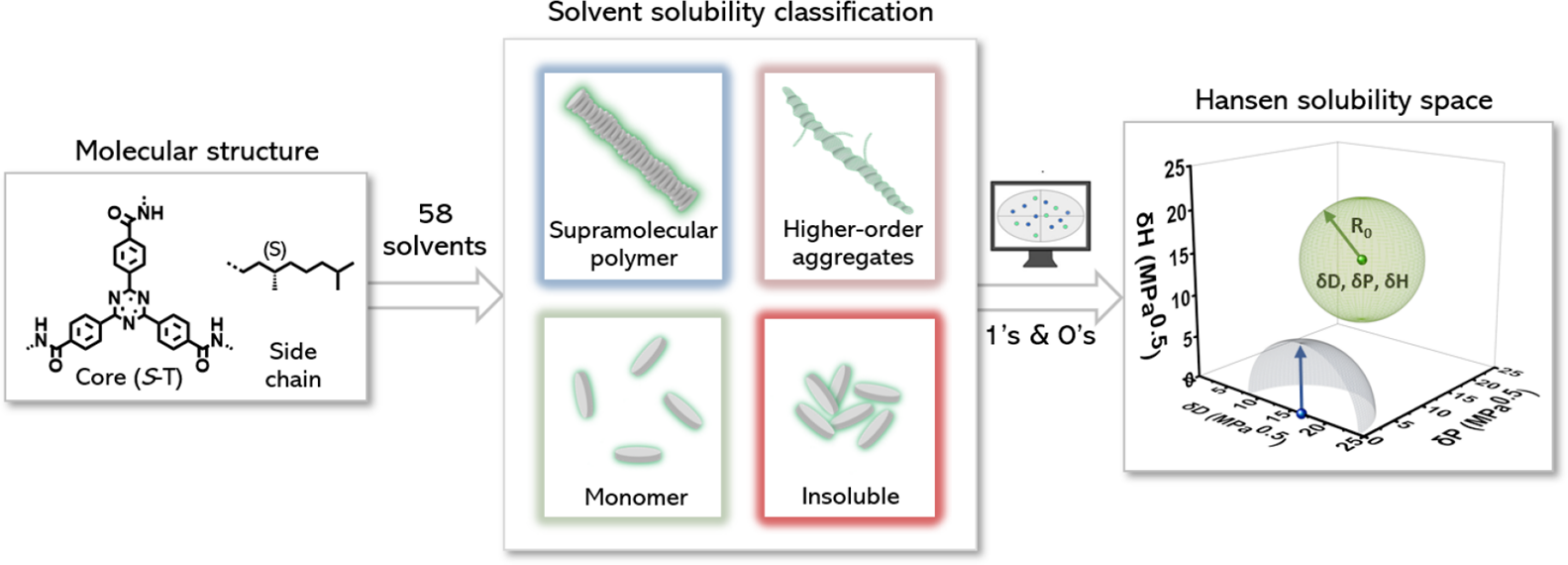
(left → right) Molecular structure of ***S*-T**, the applied solvent classification and subsequent computation of the HSPs using a dual-sphere model describing the supramolecular polymer (blue) and monomer (green) solubilities.

These preliminary results demonstrate the crucial effect of solvents on the assembly pathway of ***S*-T** and prompted us to develop a systematic parametrized approach in order to rationalize the role of solvents in supramolecular polymerization. Therefore, we employed the HSP model to describe the solubility of ***S*-T** at room temperature in 58 solvents with known HSPs^47^ covering the Hansen solubility space. Similar to the solvent study above, the solubility of ***S*-T** was first evaluated visually followed by CD and UV spectroscopy in order to distinguish between the monomeric and polymeric states. The shape and intensity of the CD signal were then used to further differentiate between supramolecular polymers and HOAs. In turn, the solvents were classified into four categories (Figure 4): supramolecular polymers (SP solvents, blue dots), higher-order aggregates (HOA solvents, closed red dots), monomers (good solvents, green dots), and insoluble globular aggregates (bad solvents, open red dots). It should be noted that when scattering was observed at higher wavelengths, the solvent received a “bad solvent” classification, as this usually indicates the presence of aggregates of a reasonable size. A detailed explanation on the solvent classification is listed in Section 4 of the Supporting Information.

In order to create the HSP spheres, 1’s and 0’s are assigned to each solvent and fed to the numerical solver developed by Díaz de los Ríos et al.^46^ This multiresponse optimization algorithm computes the central coordinates (δ_D_, δ_H_, and δ_P_) and a spherical solubility boundary, defined by the sphere’s radius (*R*_0_), based on the smallest error for any solvent in the wrong position in the Hansen solubility space. After determination of the HSP coordinates and *R*_0_, the model’s accuracy to describe the solubility data is expressed in a desirability fuction (*f*_i_) for each sphere, which improves upon approaching unity. Detailed information about the computational description of the HSPs and corresponding *R*_0_ is listed in Section 5 of the Supporting Information.

### General Solubility Trends in the Hansen Solubility Space of ***S*-T**

At first, we applied a mono-sphere model, exclusively considering bad (closed red dots) and good solvents (green dots). However, the model lacked accuracy (*f*_gs_ = 0.16; green sphere; Figure S4) to predict the solubility of ***S*-T** due to its amphiphilic nature. By employing a dual-sphere model and separately classifying both the SP state (blue sphere) and the monomeric state (green sphere), the accuracy was improved significantly (*f*_gs_ = 0.96; *f*_bs_ = 0.94; Figure S4) and applied to all following experiments.

**Figure 5:**
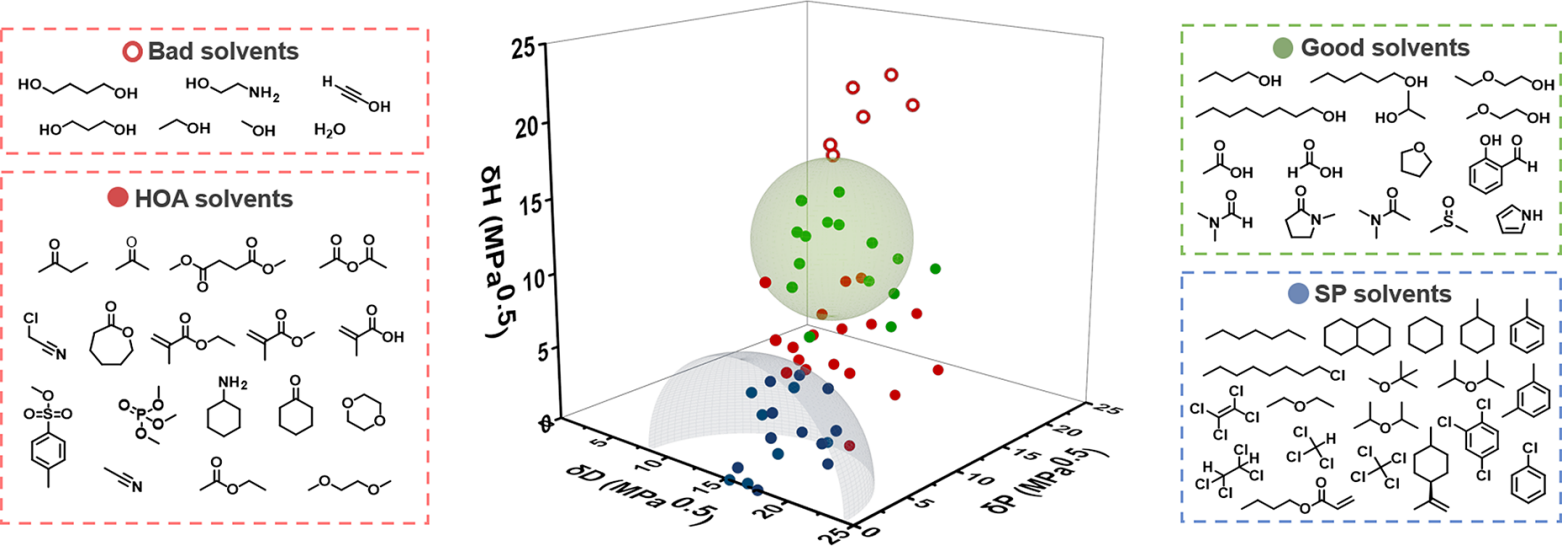
Experimentally determined 3D Hansen solubility space of ***S*-T** based on 58 different solvents. The solvents are divided into four distinct categories: bad solvents causing precipitation (open red dots), bad solvents inducing higher-order aggregates (closed red dots), good solvents providing dissolved monomers (green dots), and solvents promoting the formation of supramolecular polymers (blue dots).

By subsequent analysis of the solvent data, we perceived that δ_D_ has almost no influence on the Hansen solubility space of ***S*-T** compared to δ_H_ and δ_P_, as shown in Figure 5, suggesting that the polarity and hydrogen-bonding capability are key parameters to control polymerization. Furthermore, solvents that lack or contain either donor and/or acceptor groups appear to occupy distinct areas in the Hansen solubility space. The SP solvents that favor the supramolecular polymerization seem to predominantly consist of alkanes, ethers, and aromatic molecules lacking donor or acceptor moieties (Figure 5, blue dots; Figure S14). We assume here that the side chains of ***S*-T**, composed of solely hydrocarbons, have similar low δ_H_ and δ_P_ values as most alkanes, like heptane, therefore ensuring dissolution of the supramolecular polymer periphery. In addition, the lack of affinity with the ***S*-T** core promotes the formation of hydrogen bonds between the amides and π stacking between the aromatic moieties of ***S*-T**. The area in-between the green and blue spheres (Figure 5, closed red dots; Figure S15) consists mainly of solvents containing acceptor moieties. Surprisingly, all these solvents show comparable changes in the CD intensity and shape indicative of HOAs, which strongly suggests that the formation of HOAs is a more general phenomenon than frequently thought. We therefore postulate that the formation of an HOA originates from weak interactions between the ***S*-T** core and acceptor solvents in combination with the low affinity for the apolar side chains. Further increasing the solvent polarity and hydrogen bonding capability (larger δ_H_ and δ_P_ values) results in a region with good solvents (Figure 5, green dots; Figure S16) containing donor moieties. Considering the polar core of ***S*-T** (Figures 1 and 4), the enhanced solubility in donor solvents is ascribed to the more favorable donor–acceptor interactions between solvent and solute when compared with the rather unfavorable repulsive acceptor–acceptor interactions that dominate in the HOA solvents. This striking disparity in solubility between acceptor and donor solvents prompted us to revisit the general HSP model by splitting δ_H_ into the H-donor (δ_HD_) and H-acceptor (δ_HA_) analogues (Appendix B).^46^ Herein, the δ_D_ was replaced by either the δ_HD_ or δ_HA_, which resulted in slightly higher *f*_gs_ and *f*_bs_ values for δ_HD_, while replacement with δ_HA_ resulted in significantly lower *f*_gs_ and *f*_bs_ values (Figure S5). These outcomes further support our claim that the solvent’s ability to donate a hydrogen is crucial for both the monomer as supramolecular polymer solubility of ***S*-T** (for a detailed explanation, see Section 5.4 of the Supporting Information). Eventually, solvents with the highest δ_H_ and δ_P_ values (such as 1,3-butanediol and ethanolamine) detrimentally alter the solubility of ***S*-T** resulting in precipitation (Figure 5, open red dots). These particular solvents are able to compete with the hydrogen bonding amide groups of ***S*-T**, yet they form enthalpically unfavorable interactions due to the high δ_H_ and δ_P_ values leading to nonidentical aggregates.

Although the accuracy of the HSP model in describing the solubility of ***S*-T** is reasonable (*f*_gs_ = 0.96; Figure S4), it also shows discrepancies as indicated by the good solvents outside the green sphere: tetrahydrofuran, *N*-dimethyl formamide, *N*-methyl-2-pyrrolidone, and dimethyl sulfoxide. The three latter solvents have a relatively high dielectric constant (ε > 33), originating from a larger electron density imbalance (dipole moment) within the molecules, when compared to acceptor solvents such as esters and ketones. We postulate that the resulting partial charges induce more favorable interactions and in turn compete with the ***S*-T**/***S*-T** interactions leading to the solubilization of ***S*-T**. Furthermore, the bad solvents found inside the green sphere, such as dimethyl succinate, trimethyl phosphate, and acetic anhydride, are strong hydrogen bond acceptors with relatively high δ_H_ values. However, these acceptor solvents do not solubilize the ***S***-**T** core but promote the formation of HOAs because of unfavored repulsive acceptor–acceptor interactions, as explained in the general trend of the model. Nevertheless, the accuracy of the unoptimized model is sufficient to show rational trends that help understand solvent effects in supramolecular polymerization.

### Effect of Concentration and Temperature on the Hansen Solubility Space

**Figure 6:**
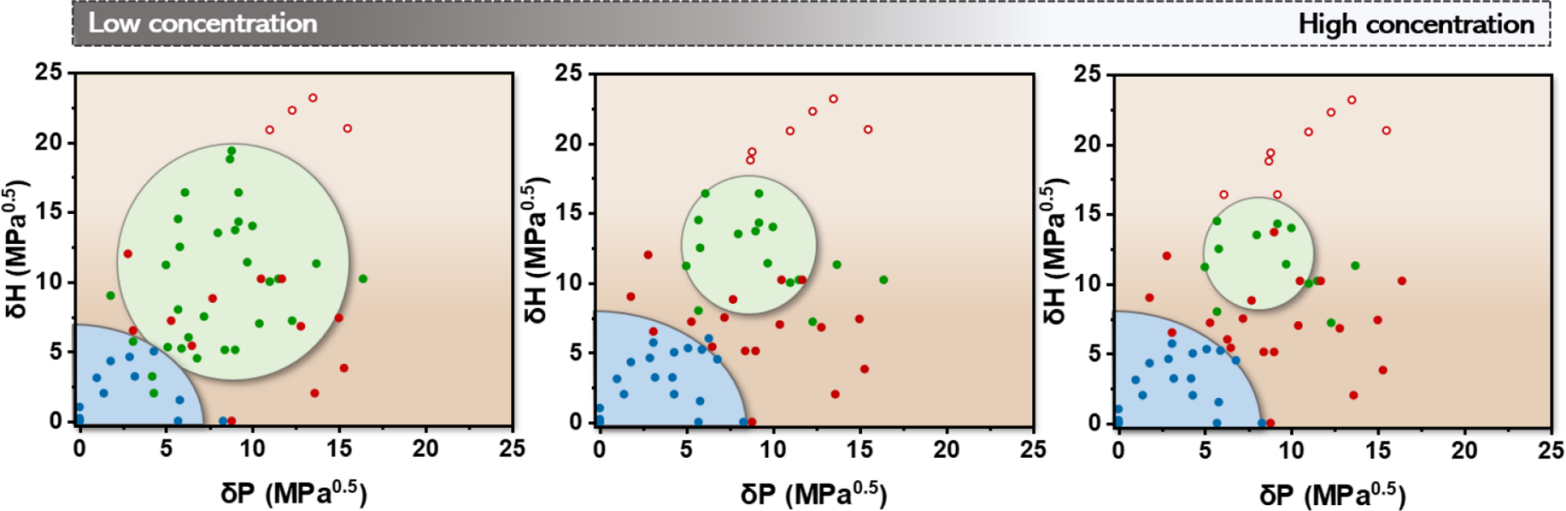
2D representation of Hansen solubility space of ***S*-T** at concentrations of 25, 100, and 500 μM (left → right) observed in the δ_H_–δ_P_ plane. Solvents are classified as bad solvents (open red dots), HOA solvents (closed red dots), good solvents (green dots), and SP solvents (blue dots).

After deriving various general solubility trends, we probed the effect of concentration on the Hansen solubility spaces for ***S*-T** (Figures 6 and S6). As shown in the 2D representation of the Hansen solubility space in Figure 6, mainly the green sphere is affected by shrinkage and expansion at higher and lower concentrations, respectively. These observations suggest that supramolecular building blocks like ***S*-T** exhibit a limited solubility in certain solvents similar to other types of compounds. On the contrary, the blue SP sphere is barely affected by the variations in concentration implying that the strong ***S*-T/*S*-T** interaction dominates in this solvent region.

Similar as to dilution, an increase in temperature induces expansion of the green sphere in all directions due to the improved solubility of monomeric ***S*-T**, while the blue sphere decreases in size because of reduced aggregation propencity (Figure S7). Herein, supramolecular polymers of ***S*-T** (blue sphere) disassemble into the monomeric state (green sphere), particularly for solvents with slightly higher δ_H_ and δ_P_ values at the edge of the blue sphere (such as chloroform and tetrachloroethane). For solvents with δ_H_ and δ_P_ values close to 0, the monomeric state is not observed in the measured temperature range (20–90 °C). Interestingly, the HOA solvent region in-between the blue and green spheres diminishes upon expansion of the green sphere at higher temperatures, which is in accordance with the temperature-dependent CD measurements of HOAs as observed in ethyl acetate (Figure 3B).

### Generality of the Model Using Structurally Different Supramolecular Monomers

**Figure 7:**
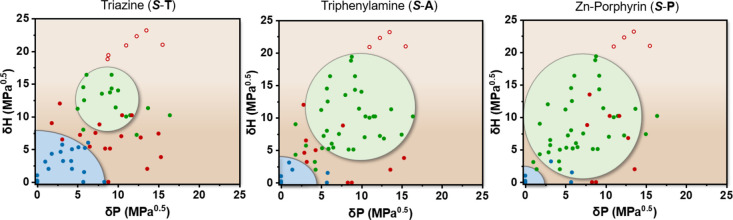
2D representation of Hansen solubility space of ***S*-T**, ***S*-A**, and ***S*-P** at 100 μM (left → right) observed from the δ_H_–δ_P_ plane. Solvents are classified as bad solvents (open red dots), HOA solvents (closed red dots), good solvents (green dots), and SP solvents (blue dots).

These detailed solubility and spectroscopical studies on ***S*-T** incited us to examine the model’s applicability on structurally different supramolecular building blocks (Figures 7 and S8). Hereto, triphenylamine (***S*-A**) was selected due to the weaker core–core interactions compared to ***S*-T**, while Zn-porphyrin (***S*-P**) was chosen due to the presence of solubilizing alkoxy wedges at the periphery. Applying the model on both molecules at room temperature results in a *f*_gs_ of 0.68 for ***S*-P** and 0.67 for ***S*-A**, while *f*_bs_ for both exceeded 0.94 (Figures S8). Compared to ***S*-T**, the green sphere of ***S*-A** expands toward solvents with lower δ_P_ and δ_H_ values. We attribute this observation to the relatively weak ***S*-A**/***S*-A** interactions enabling the HOA acceptor solvents to compete and dissolve ***S*-A**. Consequently, supramolecular polymers of ***S*-A** are only formed in the most apolar solvents resulting in a small blue sphere. For the supramolecular monomer ***S*-P**, the variations in the solubility space are comparable to ***S*-A** and consist of an expansion of the green sphere and a reduction of the blue sphere. This trend is accompanied by a shift of the green sphere toward lower δ_H_ and δ_P_ values, closing the gap between the two spheres and hence reducing the solubility region in which HOAs form. This change in coordinates is most likely caused by the increased fraction of apolar alkyl side chains in ***S*-P** and hence a stronger affinity for low δ_P_ and δ_H_ solvents. These results nicely show the central importance of supramolecular building block design in connection with the solvent used and the general applicability of the HSPs to structurally different monomers.

## Conclusions

Our study represents a parametrized solubility approach that forms a fundamental understanding on the solvent-induced pathway complexity of supramolecular building blocks. By consistent visual and spectroscopic determination of the morphological states of ***S*-T** in 58 different solvents, a solubility space was created using the Hansen solubility parameters δ_D_, δ_P_, and δ_H_. By subsequent optimization of the model and variations in concentration and temperature, we were able to acquire valuable insights into the solubility of ***S*-T** and the importance of the solvent’s ability to donate or accept hydrogen bonds on supramolecular polymerization. Besides the solvent’s selectivity for solvation of the monomer’s core or periphery, we discovered an intermediate solvent regime where HOAs are formed due to a small mismatch in polarity between the solvent and the side chains. By eventually applying the HSP model to supramolecular building blocks with different molecular structures (***S*-A** and ***S*-P**), we could demonstrate the general applicability of the model and show the crucial importance of tuning the solvent choice to the supramolecular monomer design. Vice versa, the monomer design could be tuned to be soluble in a desired solvent by developing a database, such as HSPiP, based on amphiphilic assembling building blocks. This systematic study expands our knowledge of solvent-induced pathway complexity in which interactions between the solvent, side chain, and core play a crucial role. Furthermore, these insights will help elucidate the occurrence of higher-order aggregates or single chains, which are so often reported. We propose that many other undefined HOAs observed in hydrogen-bond-based aggregation in organic solvents arise from subtle solvation effects and do not preclude the formation of well-defined individual fibers in another solvent. Although not studied here, bundled fibers are often observed when water is the solvent for supramolecular polymers and the rationale is evident from the present study. To arrive at single supramolecular polymers, the design of the periphery should make the solubility in water as high as possible. We envision that this work will further advance the development of complex functional supramolecular systems and materials.

## Supporting Information Available

Materials and methods; synthetic procedures; sample preparation; solvent classification; computational methods and three-dimensional solubility details; additional AFM images; supporting UV–vis/CD spectra; HSP parameters; and solvent data (PDF)
