# Supplementary material for: Solvent-Induced Pathway Complexity of Supramolecular Polymerization Unveiled Using the Hansen Solubility Parameters
Source: J Am Chem Soc. 2023 Aug 2;145(32):17987–94. doi: 10.1021/jacs.3c05547 (PMC10436269; doi:10.1021/jacs.3c05547)
Supplement: Supplementary file 1 — ja3c05547_si_001.pdf [file ja3c05547_si_001.pdf]

Supporting information

Solvent-Induced Pathway Complexity of Supramolecular Polymerization Unveiled Using the Hansen Solubility Parameters

Joost J. B. v. d. Tol[1]*, Ghislaine Vantomme*[1]** and E. W. Meijer*[1,2]**.*

[1] *Institute for Complex Molecular Systems and Laboratory of Macromolecular and Organic Chemistry, Eindhoven University of Technology, P.O. Box 513, 5600 MB Eindhoven, The Netherlands.* [2] *School of Chemistry and RNA Institute the University of New South Wales, Sydney, NSW 2052, Australia*.

Table of Contents

1. Materials and methods S2
2. Synthesis of *S*-T, *S*-A and *S*-P S3
3. Sample preparation and preliminary solvent studies S5
4. Solvent classification workflow S6
5. A comparison between the mono- and dual sphere model and the δ_{HA} and δ_{HD} S7
6. Effect of concentration, temperature and molecular design on the 3D Hansen solubility space. S12
7. Atomic Force Microscopy measurements S15
8. Circular dichroism spectra of *S*-T, *S*-A and *S*-P at 100 µM. S17
9. References S20

Appendix S21

1. Materials and Methods

S1.1 Materials

The supramolecular building blocks ***S*-T**, ***S*-P** and ***S*-A** were obtained from previously prepared batches.[1–3] All solvents were purchased from commercial resources and purified by passing through a basic aluminium plug and drying over 4 Å molsieves for at least 24 hours before use. Deuterated solvents were obtained from Cambridge Isotopes Laboratories.

S1.2 Methods

NMR spectra were recorded using a Varian Mercury Vx 400 MHz (^{1}H-NMR using 400 MHz and ^{13}C-NMR using 100 MHz). Proton and carbon chemical shifts are reported in ppm (δ) downfield from tetramethylsilane (TMS) using the deuterated solvent resonance frequency as internal standard. Peak multiplicities are abbreviated as s: singlet; d: doublet; t: triplet; q: quartet; p: pentet; m: multiplet; dd: double doublet; dt: double triplet and dq: double quartet.

UV/Vis and circular dichroism (CD) measurements were performed on a JASCO J-815 CD spectrometer, equipped with either a JASCO Peltier MPTC-490S temperature controller (temperature range of 278 – 373 K) or a JASCO Peltier PFD-425S/15 (temperature range of 263 – 383 K), and a Jasco FMO-427S/15 emission monochromator. Hellma Quartz Suprasil cuvettes with an optical pathlength of 0.1 cm were used for all spectroscopic measurements.

Atomic Force Microscopy (AFM) studies were conducted using a Cypher Environmental Scanner (ES) equipped with a closed cell and a heating and cooling stage. Silicon probes (NCSTR) with a tip height of 10–15 µm, radius of 7 nm, frequency of 160 kHz and a force constant of 7.4 N/m were used to record images in tapping-mode (phase<90). Furthermore, a scan rate of ~2 Hz and a resolution of 1024x1024 pixels were used. Samples were prepared by dropcasting 20 µL of 100 µM solutions of ***S*-T** from the desired solvent onto freshly cleaved 1x1 cm^2 sized mica followed by overnight drying in air. Contrast of the images was further enhanced using first order planefit and flattening using Gwyddion v2.60.

2. Synthesis of the molecules used in the study *S*-T, *S*-A and *S*-P

S2.1 Synthesis of 4,4',4''-(1,3,5-triazine-2,4,6-triyl)tris(N-((S)-3,7-dimethyloctyl) benzamide) (S-T)

A previously synthesized batch of chiral *S*-triazine (***S*-T**), from which the synthesis has been published before,[1] was used for all experiments.

^{1}H NMR (400 MHz, Chloroform-d_1 and 3 vol% TFA- d_1): δ [ppm] = 8.57 (s, 6H), 7.79 (s, 6H), 3.57 (s, 6H), 1.74 (s, 3H), 1.64 – 1.48 (m, 9H), 1.41 – 1.11 (m, 18H), 0.99 (d, ^{3}J = 5.6 Hz, 9H), 0.89 (d, ^{3}J = 6.5 Hz, 18H). ^{13}C NMR (101 MHz, Chloroform-d_1 and 3 vol% TFA-d_1): δ [ppm] = 170.70, 169.80, 138.95, 136.49, 129.54, 127.53, 39.65, 39.34, 37.22, 36.28, 31.06, 28.12, 24.81, 22.78, 22.68, 19.50. MALDI-TOF-MS found: 859.31 m/z (calculated [M+H]$^{+}$: 859,61). FT-IR (cm^{-1}): 3256, 2956, 2923, 2870, 1631, 1579, 1545, 1517, 1467, 1406, 1370, 1313, 1259, 1145, 1099, 1018, 869, 821, 742, 698, 677, 631, 532.

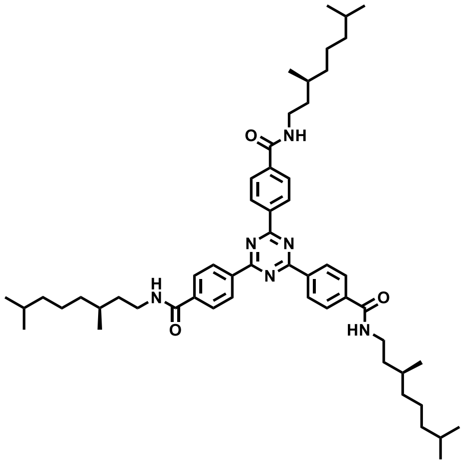

S2.2 Synthesis of 4,4',4''-nitrilotris(N-((S)-3,7-dimethyloctyl)benzamide) (S-A)

A previously synthesized batch of chiral *S*-triphenylamine (***S*-A**), from which the synthesis has been published before,[2,4] was used for all experiments.

^{1}H NMR (400 MHz, Chloroform-d_1): δ [ppm] = 7.67 (d, 6H, ^{3}J = 16 Hz), 7.10 (d, 6H, ^{3}J = 16 Hz), 5.98 (t, 3H, ^{3}J = 10 Hz), 3.50-3.43 (m, 6H), 1.66-1.13 (m, 36H), 0.94 (d, 9H, ^{3}J = 12 Hz), 0.86 (d, 18H, ^{3}J = 16 Hz). ^{13}C NMR (101 MHz, Chloroform-d_1) δ [ppm]: 166.65, 149,26, 129,95, 128,33, 123.90, 39.23, 38.27, 37.81, 36.81, 30.81, 27.95, 24.66, 22.70, 22.60, 19.27. MALDI-TOF-MS found: 795.62 m/z (calculated for [M+H]$^{+}$: 795.61). FT-IR (cm^{-1}): 3311, 3071, 2953, 2926, 2868, 1632, 1599, 1548, 1499, 1499, 1467, 1379, 1366, 1297, 1280, 1187, 1146, 1111, 1016, 959, 847, 766, 734, 681, 662, 627, 537.

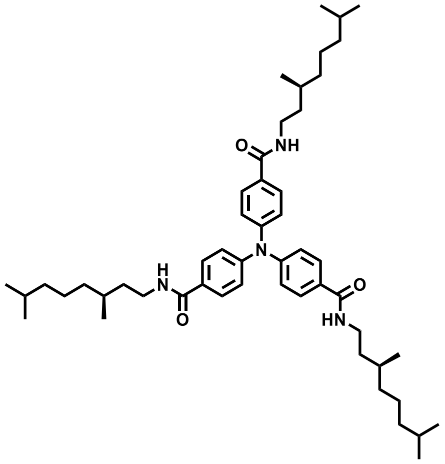

S2.3 Synthesis of 4,4',4''-(1,3,5-triazine-2,4,6-triyl)tris(N-((S)-3,7-dimethyloctyl) benzamide) (S-*P*)

A previously synthesized batch of chiral Zn inserted *S*-Porphyrin (***S*-P**),[3,5] from which the synthesis has been published before,[6] was used for all experiments.

^{1}H-NMR (400 MHz, Chloroform-d_1): No inner pyrrole NH observed at $\delta < 0$ ppm after Zn-insertion, δ [ppm] = 0.78-0.91 (m, 72H), 0.92-1.01 (m, 36H), 1.12-2.04 (m, 120H), 3.96-4.16 (m, 24H), 7.08 (s, 8H), 8.05 (s, 4H), 8.25 (d, ^3J = 8 Hz, 8H), 8.35 (d, ^3J = 8 Hz, 8H), 8.96 (s, 8H, β-pyrrole). ^{13}C-NMR (100 MHz, Chloroform-d_1): δ 19.8, 22.8, 22.9, 24.9, 28.1, 29.9, 30.0, 36.6, 37.5, 37.7, 39.4, 39.5, 67.6, 72.0, 99.5, 120.2, 125.4, 132.2, 133.7, 134.3, 134.8, 135.4, 146.5, 150.0, 153.5, 165.8. MALDI-TOF-MS found: 3026.04 m/z (calculated for $[M+H]^+$: 3026.13). FT-IR (cm^{-1}): 3302, 1646, 1602.

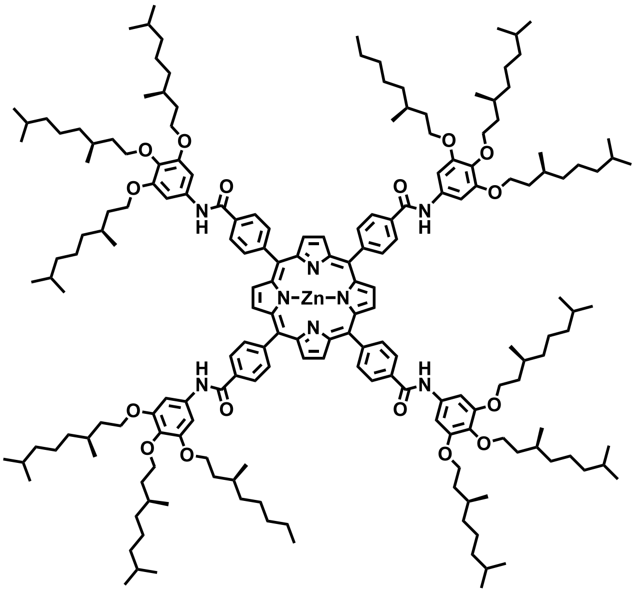

3. Sample preparation and preliminary solvent studies

S3.1 Sample preparation of all samples for CD measurements.

Stock solutions of 1 mM ***S*-T**, ***S*-P** and ***S*-A** were prepared by weighing the desired amount of compound in a 20 mL vial. For ***S*-T**, diethyl ether was added as a solvent and dichloromethane for ***S*-P** and ***S*-A** using Gilson MICROMAN positive displacement pipettes. Subsequently, 25, 100 or 500 µL of each stock solution was added to 58 different 2.5 mL vials and dried overnight under ambient conditions. Afterwards, 1 mL of each solvent dried over molecular sieves, listed in Appendix A, was added to the corresponding vial followed by heating to the solvent's boiling point and vortexing for 15 seconds. After subsequent cooling to 20 °C, the samples were equilibrated for at least 30 minutes before each visual classification of the solvent or CD measurement. All CD and UV measurements were performed with freshly prepared solutions up to maximum 1 month after preparation to minimize the contamination with water.

S3.2 CD Cooling curves of S-T in 4 different solvents; preliminary solvent studies.

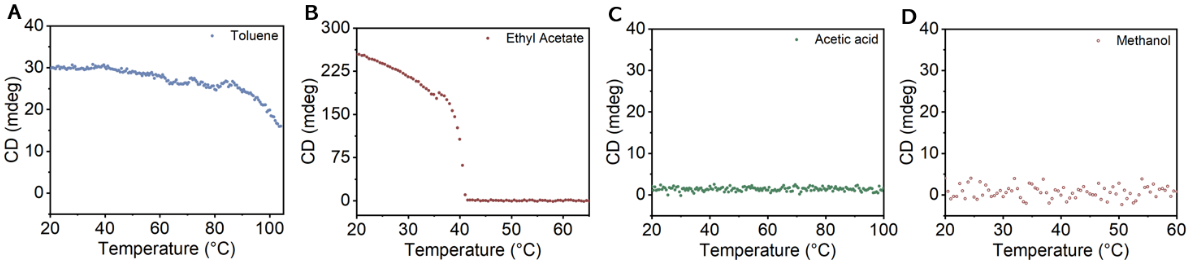

Figure S1: CD cooling curves of 100 µM solutions of ***S*-T** in (A) toluene, (B) ethyl acetate, (C) acetic acid and (D) methanol. All solutions are cooled with 1 °C min^{-1} from ~5 °C under the corresponding boiling point to 20 °C. The CD cooling curve of toluene was followed at 288 nm, in contrast to ethyl acetate, which was followed at 296 nm due to the distinct CD maximum of the coiled HOA morphology. Acetic acid and methanol did not show any change by either following the CD at 288 or 296 nm. An estimate of the T_e for toluene was made by overlapping the concentration corrected cooling curves of 25 µM and 100 µM solutions.

S3.3 Cartoon of the assembly process.

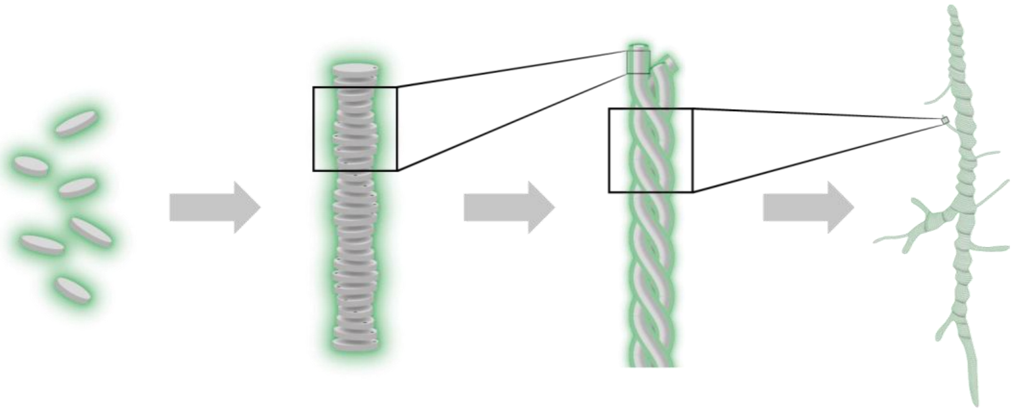

Figure S2: Cartoon of the hierarchical assembly of ***S*-T** into higher-order aggregates. A similar phenomenon was observed for ***S*-P** (Figure S13B).

4. Solvent classification workflow

S4.1 Solvent classification rationale.

After preparation of the samples as described above, the solutions were subjected to a systematic solvent classification workflow as shown in Figure S3 below. Important to consider when categorizing the solvents is the 'amphiphilic' nature of the supramolecular building blocks. As illustrated in the preliminary solvent study, their amphiphilic character gives rise to two solubilized morphologies, namely, supramolecular polymers and monomers. In case of supramolecular polymers, only the apolar aliphatic side chains are solubilized and promote hydrogen bonding and π stacking. In the latter example, the solvents are able to form favourable interactions with the ***S*-T** core and compete with the intermolecular interactions leading to the monomeric state. Additionally, two non-solubilized morphologies were formed: higher-order aggregates (HOAs) and globular aggregates. In the former case, the solvents are not able to compete with the ***S*-T/ *S*-T** interactions in combination with a low affinity for the apolar side chains, which induces agglomeration of the supramolecular polymers into HOAs. For the globular aggregates, the solvents largely form unfavourable interactions with ***S*-T** due to their very high δ_P values, which eventually leads to precipitation. These observations and postulations are discussed in more detail in the paragraph "general solubility trends in the Hansen solubility space of ***S*-T**" in the main text.

S4.2 Experimental workflow for solvent classification.

First, the samples were inspected on precipitate formation and divided into two types of categories: fibrous and globular aggregates. The solvent was classified as a 'bad solvent' if globular aggregates were observed and marked as open red dots in the Hansen solubility space. Samples containing fibrous aggregates were checked for a mono-signate CD signal to verify the presence of higher-order aggregates (classified as 'HOA solvents' and marked as closed red dots). For visual determination of the precipitates smaller than visible by eye, a green laser was used via scattered light. If a clear solution was obtained, spectroscopic measurements (CD and UV) were used to determine if the supramolecular building blocks formed supramolecular polymers or were molecularly dissolved. In the absence of a clear Cotton effect, the solvent was classified as a 'good' solvent and indicated with a green colour in the Hansen solubility space. It should be noted that if a raised baseline was observed, which is generally indicative of scattering, the solvent was classified as a 'bad solvent'. If a clear Cotton effect was present, the solvent was classified as a 'supramolecular polymer solvent' or 'SP solvent' indicated with a blue colour.

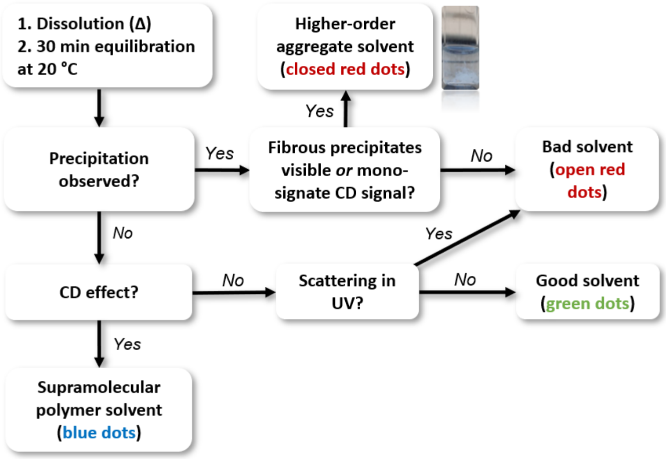

Figure S3: General systematic workflow used for the solvent classification of 100 µM solutions of ***S*-T**, ***S*-P** and ***S*-A**.

5. A comparison between the mono- and dual sphere model and the δ_{HA} and δ_{HD}.

S5.1 Mono- and dual sphere solvent assignation.

From the obtained solvent classification data, we could calculate the unknown HSPs and corresponding R_0 of the supramolecular building blocks. As previously described, all solvents are divided into four categories: SP solvents, good solvents, HOA solvents and bad solvents. For the calculation of the unknown HSPs, we utilized a multiresponse optimization problem algorithm in Excel developed by de los Rios et al.[7], which requires 1's and 0's in combination with the known HSPs of the solvents as input. For the mono sphere model, simply consisting of the sphere representing the molecularly dissolved state (green), all good solvents are assigned with 1's and all bad, HOA and SP solvents with 0's.

Applying a dual sphere model requires an additional separate solvent assignation, which represents the solubility of the side chains and thus supramolecular polymer state (blue sphere). Herein, the green sphere is based on 1's for all good solvents and 0's for all bad, HOA and SP solvents, while the blue sphere is based on 1's for all SP solvents and 0's for all good, HOA and bad solvents.

S5.2 Calculation of the Hansen Solubility Parameters, model assumptions and the desirability function.

After assignation of 1's and 0's, the algorithm tries to fit all data (known HSPs of solvents) into a 3-dimensional spherically shaped space with a radius R_0 in which the central point represents the unknown HSPs of the supramolecular building block. For the green sphere, describing the molecularly dissolved state, the following limitations apply to the algorithm: δ_D should be higher than 13 $MPa^{0.5}$; both δ_H and δ_P should be larger than 0 $MPa^{0.5}$ and R_0 should be in-between 1 and 12 $MPa^{0.5}$. For the blue sphere, essentially describing the solubility of the "alkane"-like side chains, we assume that both δ_H and δ_P should be 0 $MPa^{0.5}$, since most alkane solvents exhibit similar δ_H and δ_P values. Furthermore, δ_D should be higher than 13 $MPa^{0.5}$ and R_0 should be in-between 1 and 12 $MPa^{0.5}$. It should be noted that all 3D solubility data are presented in a non-classical way for clarity and with the reason to simplify the graphical representation. This means that the δ_D axis is not multiplied with a factor of '2', which does not affect the presented 3D solubility data.

To optimize the model for the 100 µM solutions of ***S*-T**, we excluded as many 'bad solvents' as possible from the green sphere. Therefore, the following 'good' solvents are considered as outliers and classified with a "0": tetrahydrofuran (THF), N-methyl pyrrolidone (NMP), N-dimethyl formamide (DMF), and dimethyl sulfoxide (DMSO). Interestingly, all these outliers except for THF exhibit a high dielectric constant compared to other acceptor solvents enabling these solvents to compete with the ***S*-T**/***S*-T** interactions. For the 25 and 500 µM solutions of ***S*-T** and 100 µM solutions of ***S*-P** and ***S*-A** no outliers were considered.

The quality of the model's fit is expressed via a 'desirability function' exhibiting values between 0 and 1. The desirability function is defined as follows:

$$Desirability\ function = \sqrt[n]{\prod_{i=1}^{n} f_i}$$

in which n represents the number of solvents used and f_i is described as: $f_i = e^{+(R_0 - R_a)}$ for misplaced solvents outside of the sphere ($R_a > R_0$); $f_i = e^{+(R_a - R_0)}$ for misplaced solvents inside the sphere ($R_a < R_0$) and $f_i = 1$ for correctly situated solvents outside and inside the sphere. The smaller the errors for any solvent in the wrong position in the HSP space, the higher the value of f_i (approaching unity), the better the fit. The goodness-of-fit deteriorates if f_i decreases and approaches 0, vice versa.

S5.3 mono- vs dual sphere model for supramolecular building blocks.

At first, we applied a mono sphere model, exclusively considering bad solvents (red dots) and good solvents (green dots). However, the model appeared to be incompatible with the amphiphilic nature of ***S*-T** resulting in a low f_i of 0.16 (Figure S4, left). However, by applying a dual sphere model and separately classifying both the supramolecular polymer state (blue sphere, blue dots) and the molecularly dissolved state (green sphere), f_i could be significantly improved to 0.94 and 0.96, respectively (Figure S3, right). This significant increase in accuracy motivated us to apply the dual sphere model to all different conditions in this study.

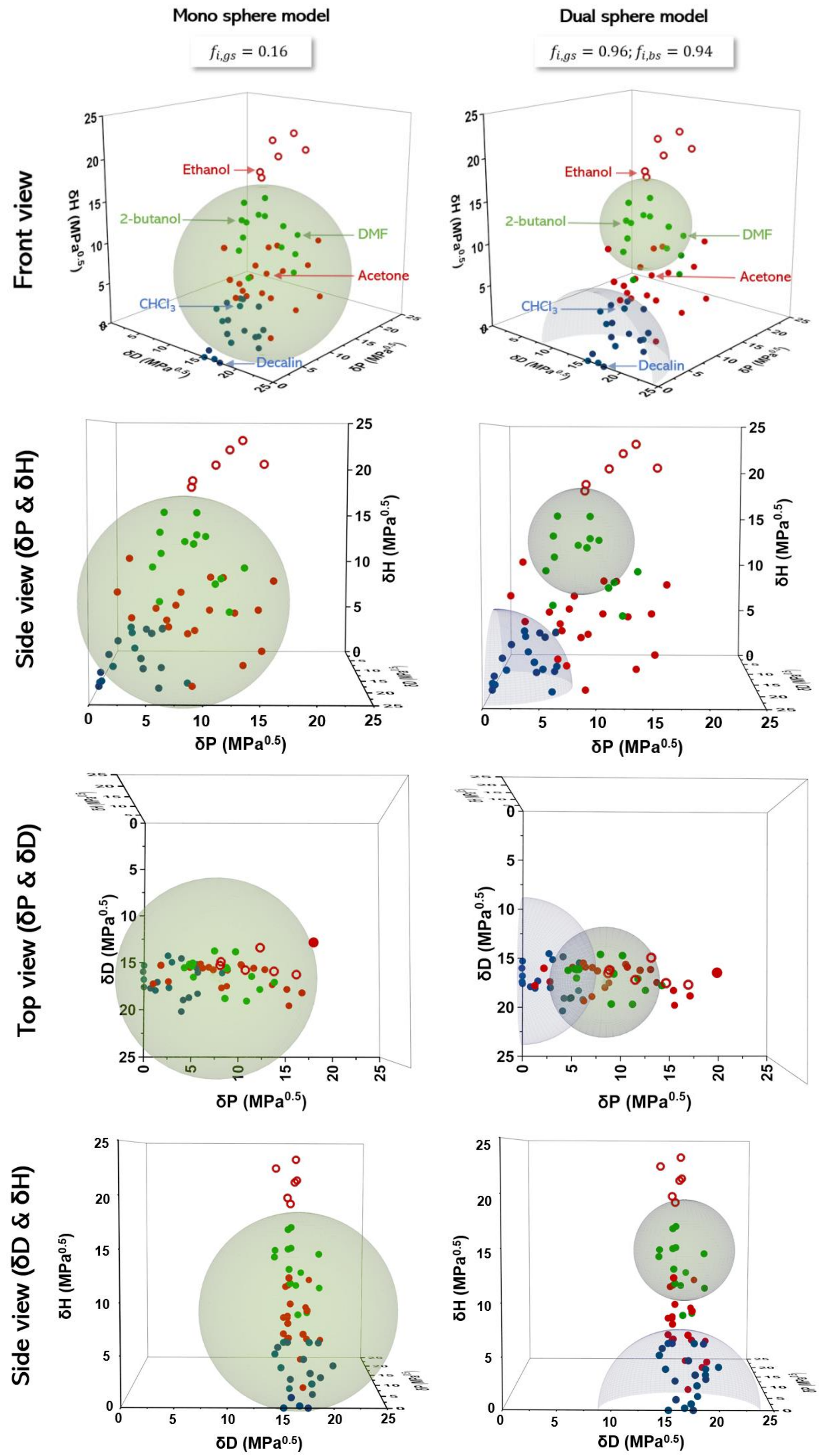

Figure S4: 3D Hansen solubility spaces of 100 µM ***S*-T** solutions for a mono sphere model and dual sphere model with corresponding $f_{i,gs}$ and $f_{i,bs}$ (left → right). The solubility spaces are displayed from the front, the $\delta_P - \delta_H$ plane, the $\delta_P - \delta_D$ plane and the $\delta_H - \delta_D$ plane (top → bottom).

S5.4 Comparison of HSP models including the H-donor and H-acceptor parameters.

The general Hansen Solubility model merely describes the ability of a molecule to form H-bonds and thus does not consider interactions between donor and acceptor moieties. In some cases, however, this distinction is crucial for describing the solubility of a compound since acceptor moieties interact more strongly with donor moieties than with themselves. As shown in Figure 4, a clear difference in solubility between acceptor and donor solvents was observed for ***S*-T**, which incited us to revisit the general HSP model by splitting δ_{H} into the H-donor (δ_{HD}) and H-acceptor (δ_{HA}) analogues.[8] Taking inspiration from M. J. Louwerse et al.[8] and C.A. Hunter,[9] we calculated the hydrogen bond acceptor parameter (δ_{HA}) and hydrogen bond donor parameter (δ_{HD}) for all 58 solvents used in this study (Appendix B). Subsequently, the influence of both parameters was determined by replacement of the δ_{D} with either δ_{HA} or δ_{HD} in our HSP model followed by a critical comparison of the f_i (Figure S5). In this particular case, the δ_{H}, δ_{P} and δ_{HD} or δ_{HA} should be larger than 0 $MPa^{0.5}$ and R_0 should be in-between 1 and 12 $MPa^{0.5}$ for the green sphere. For the blue sphere, all three parameters were assumed to be 0 $MPa^{0.5}$ with R_0 in-between 1 and 12 $MPa^{0.5}$.

As shown in Figure S5, replacement of δ_{D} by δ_{HD} slightly enhanced the $f_{i,gs}$ from 0.96 to 0.98 and $f_{i,bs}$ from 0.94 to 0.96. But more importantly, $f_{i,gs}$ and $f_{i,bs}$ declined to 0.91 and 0.74 upon replacement of δ_{D} by δ_{HA}, respectively. This observation further supports our claim that the solvent's ability to donate a hydrogen is more relevant for both the monomer as supramolecular polymer solubility of ***S*-T** than the ability to accept a hydrogen.

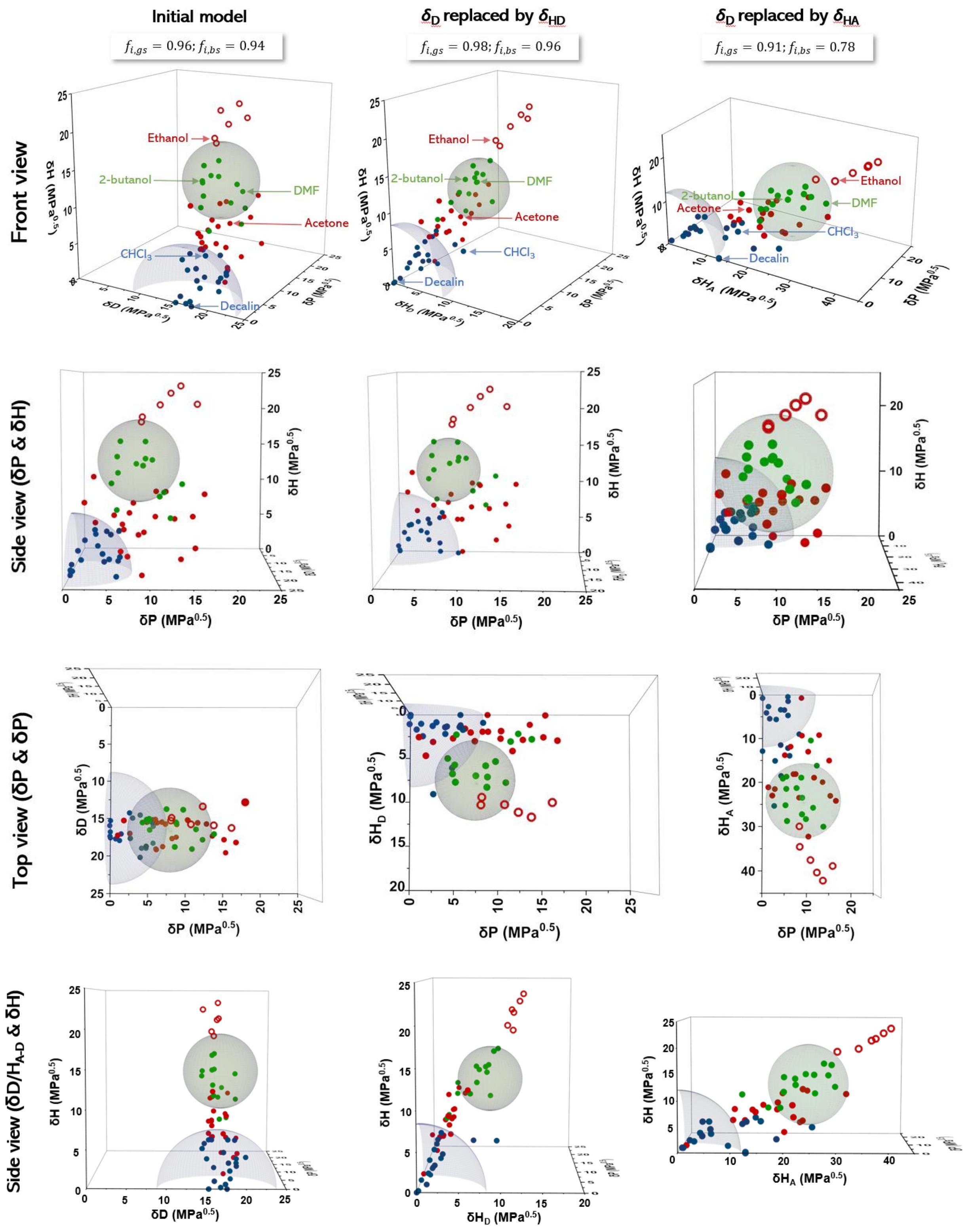

Figure S5: 3D Hansen solubility spaces of 100 µM **S-T** solutions for the conventional HSP model including δ_{HD} and δ_{HA} with corresponding $f_{i,gs}$ and $f_{i,bs}$ (left → right). The solubility spaces are displayed from the front, the $\delta_P - \delta_H$ plane, the $\delta_P - \delta_D$ plane and the $\delta_H - \delta_D$ plane (top → bottom).

6. Effect of concentration, temperature, and molecular design on the 3D Hansen solubility space.

*S6.1 3D Hansen solubility spaces of S-*T *as function of concentration.*

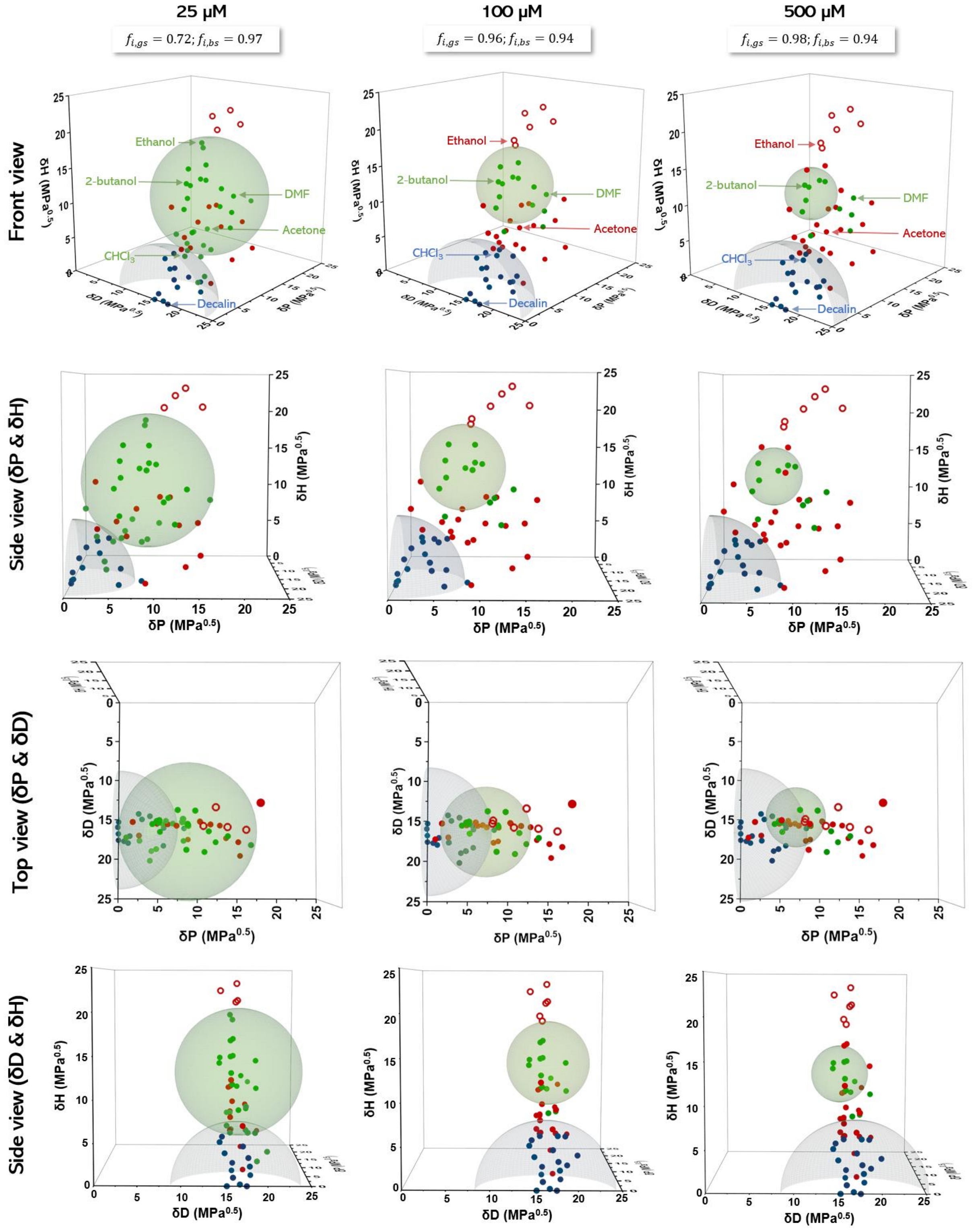

Figure S6: 3D Hansen solubility spaces of **S-T** at 25, 100 and 500 µM with corresponding $f_{i,gs}$ and $f_{i,bs}$ (left → right). The solubility spaces are displayed from the front, the $\delta_P - \delta_H$ plane, the $\delta_P - \delta_D$ plane and the $\delta_H - \delta_D$ plane (top → bottom).

S6.2 3D Hansen solubility spaces of S-T at different temperatures.

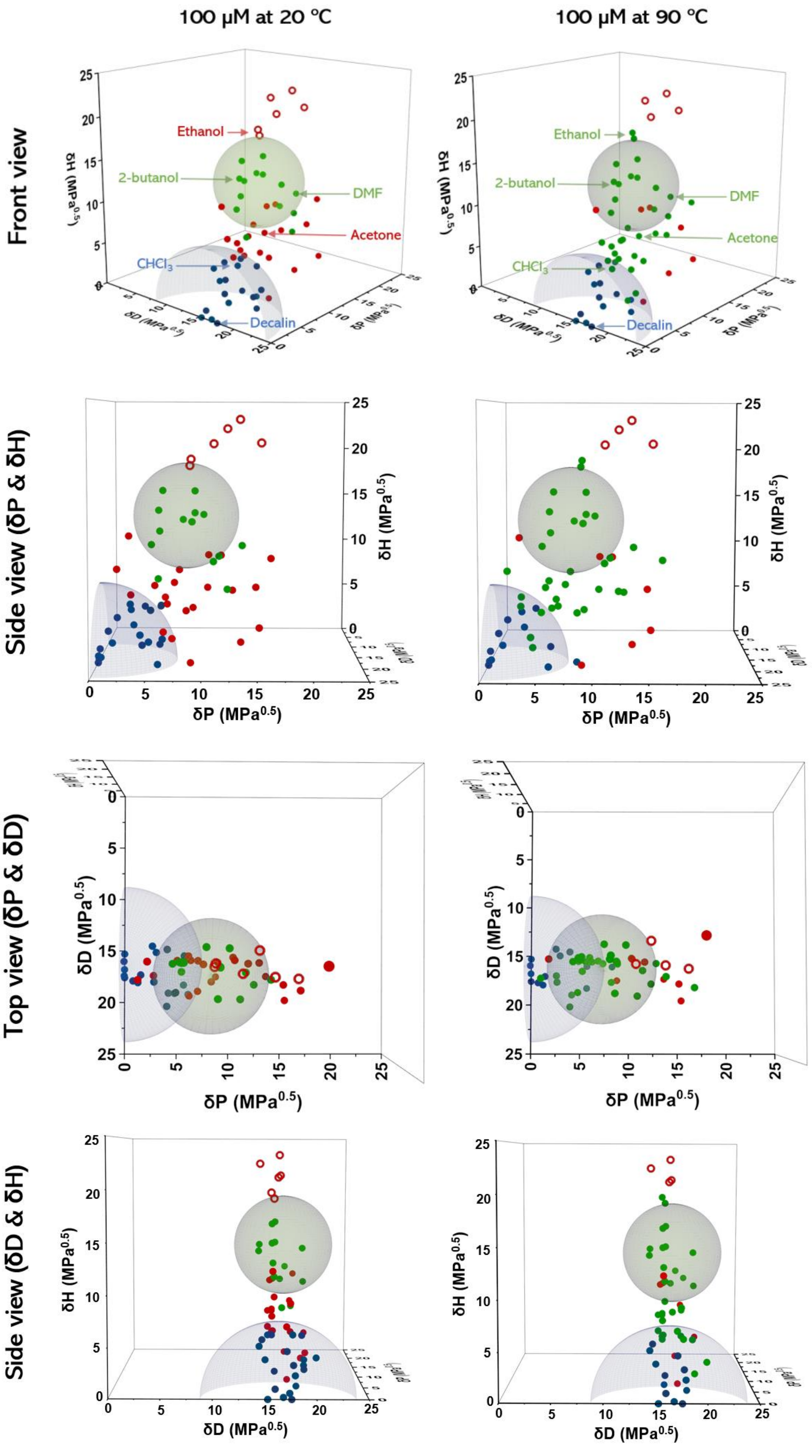

Figure S7: 3D Hansen solubility spaces of 100 µM solutions of ***S*-T** at 20 °C and 90 °C (left → right). The solubility spaces are displayed from the front, the $\delta_P - \delta_H$ plane, the $\delta_P - \delta_D$ plane and the $\delta_H - \delta_D$ plane (top → bottom).

*S6.3 3D Hansen solubility spaces of **S-P** and **S-A**.*

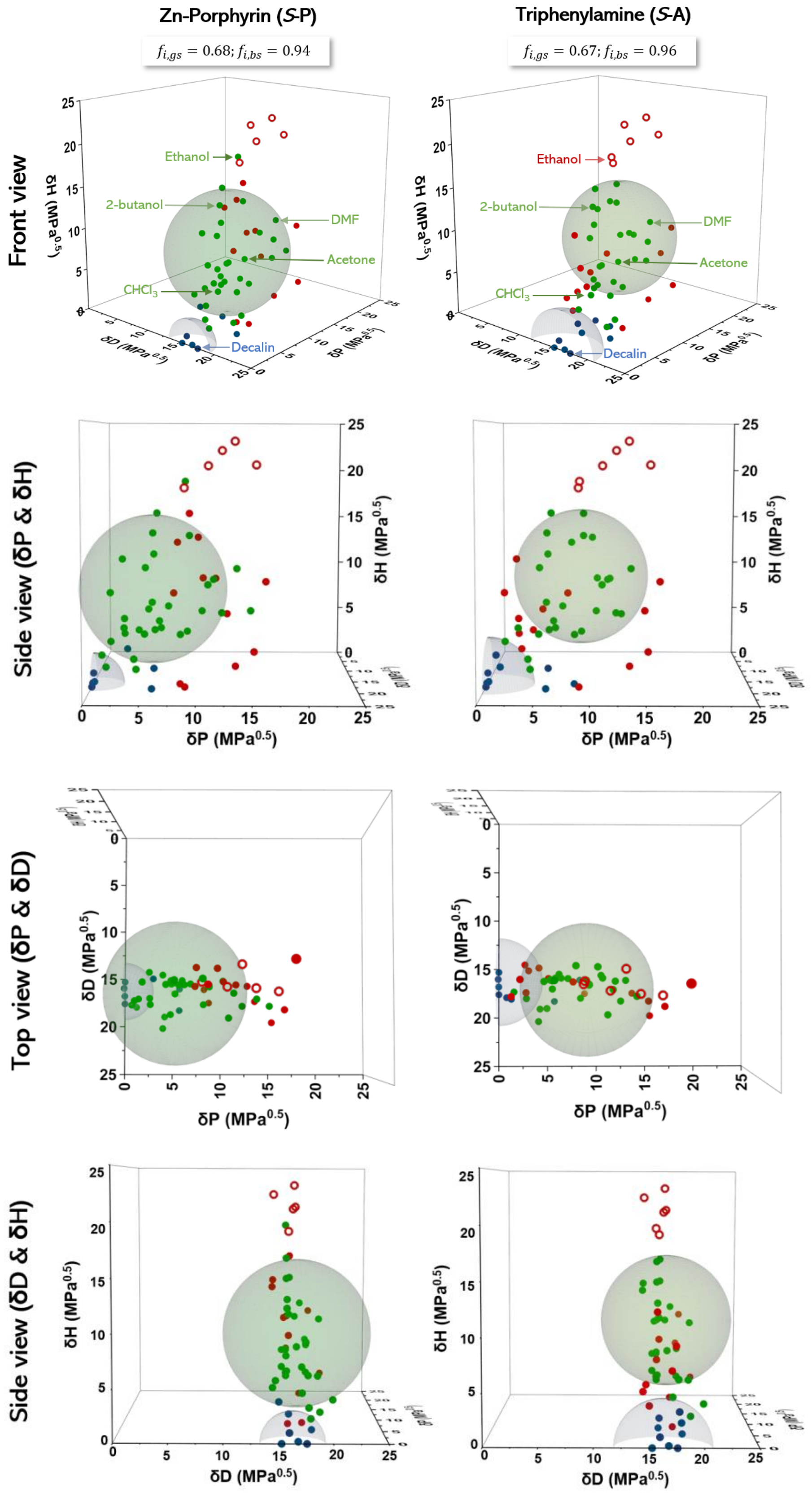

Figure S8: 3D Hansen solubility spaces of 100 µM solutions of ***S*-P** and ***S*-A** with corresponding $f_{i,gs}$ and $f_{i,bs}$ (left → right). The solubility spaces are displayed from the front, the $\delta_P - \delta_H$ plane, the $\delta_P - \delta_D$ plane and the $\delta_H - \delta_D$ plane (top → bottom).

7. Atomic Force Microscopy measurements

S7.1 Atomic force microscopy images of S-*T *at from various solvents.

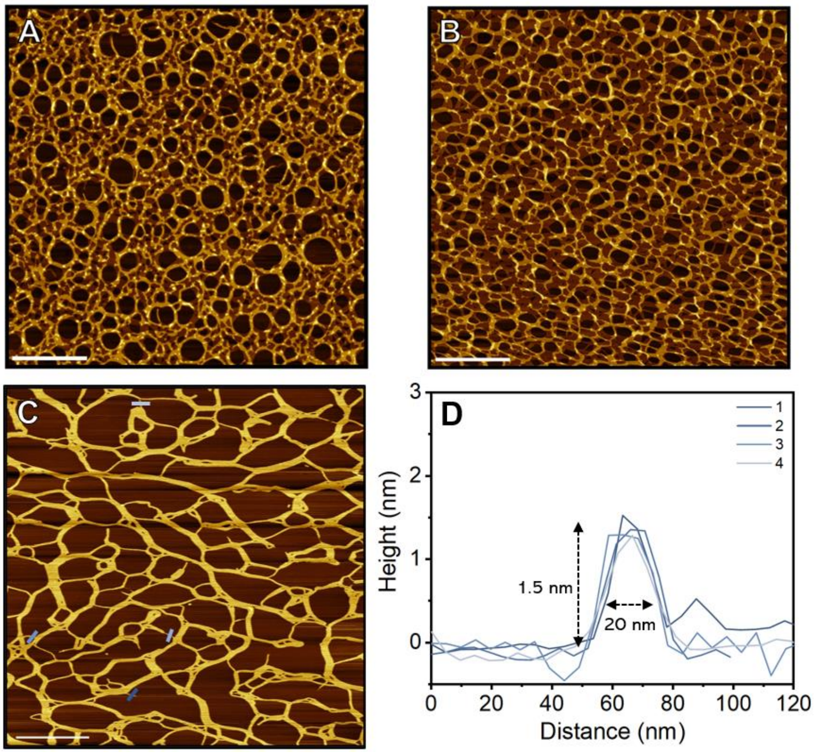

Figure S9: 10x10 µm AFM height images of dropcasted samples (20 µL) of 100 µM ***S*-T** solution from (A) cyclohexane and (B) toluene. The inset scalebar represents 2 µm. (C) 5x5 µm AFM height images of spincoated samples (20 µL) of 100 µM ***S*-T** solution from toluene. The inset scalebar represents 1 µm. (D) Corresponding height profile of the assemblies of ***S*-T** along the lines as indicated in Figure 9C, illustrating the presence of single supramolecular polymers.

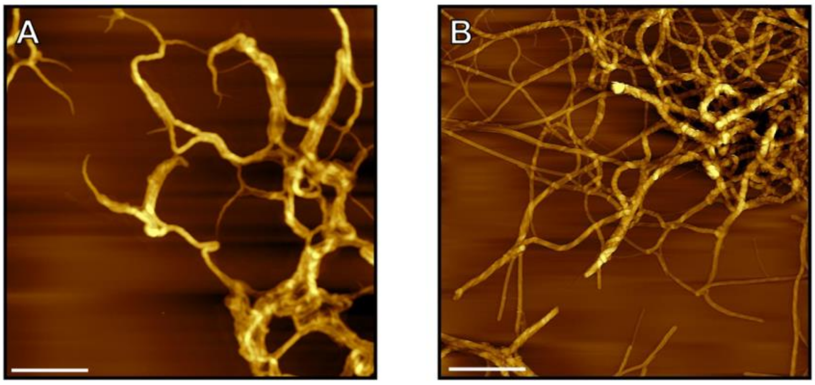

Figure S10: 10x10 µm AFM height images of dropcasted samples (20 µL) of 100 µM ***S*-T** solution from (A) acetone and (B) ethyl acetate. The inset scalebar represents 2 µM.

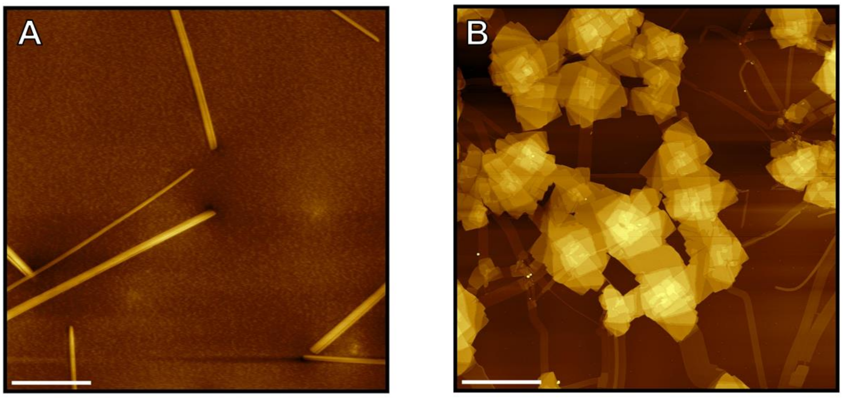

Figure S11: 10x10 µm AFM height images of dropcasted samples (20 µL) of 100 µM ***S*-T** solution from (A) acetic acid and (B) dimethyl formamide. The inset scalebar represents 2 µm.

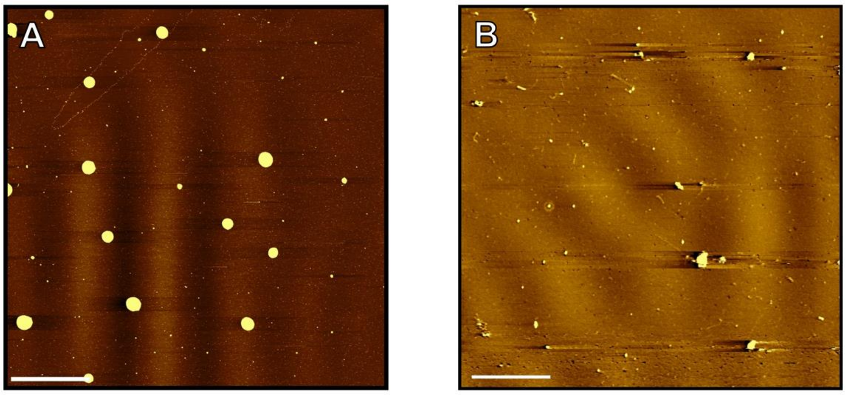

Figure S12: 10x10 µm AFM height images of dropcasted samples (20 µL) of 100 µM ***S*-T** solution from (A) methanol and (B) water. The inset scalebar represents 2 µm.

S7.2 Atomic force microscopy images of S-*A *and S-*P *from higher-order aggregate solvents.

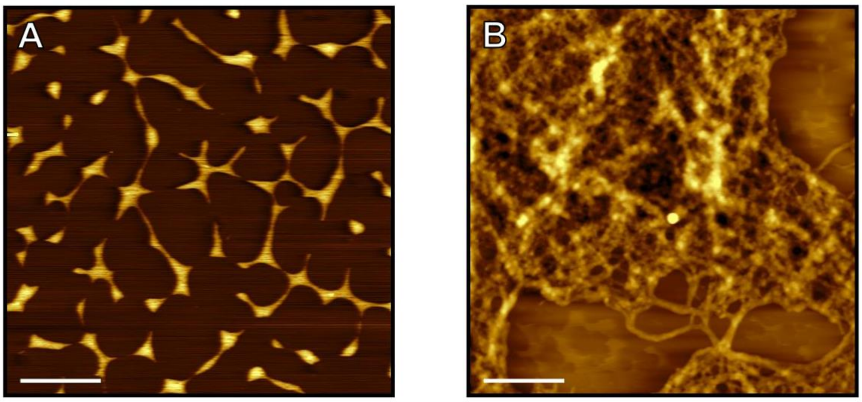

Figure S13: 10x10 µm AFM height images of dropcasted samples (20 µL) of 100 µM (A) ***S*-A** from t-butyl methyl ether and (B) ***S*-P** from dimethyl succinate (higher-order aggregate solvents). The inset scalebar represents 2 µm.

8. Circular dichroism spectra of *S*-T, *S*-A and *S*-P at 100 µM.

S8.1 CD spectra of 100 µM solutions of S-T in SP, HOA and good solvents.

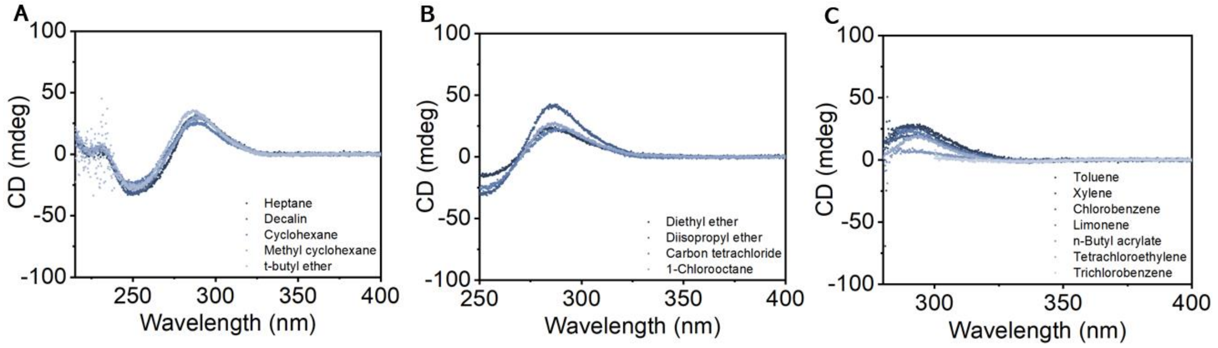

Figure S14: CD spectra of 100 µM solutions of ***S*-T** in SP solvents grouped for solvent cut-off below (A) 220 nm, (B) 250 nm and (C) 280 nm.

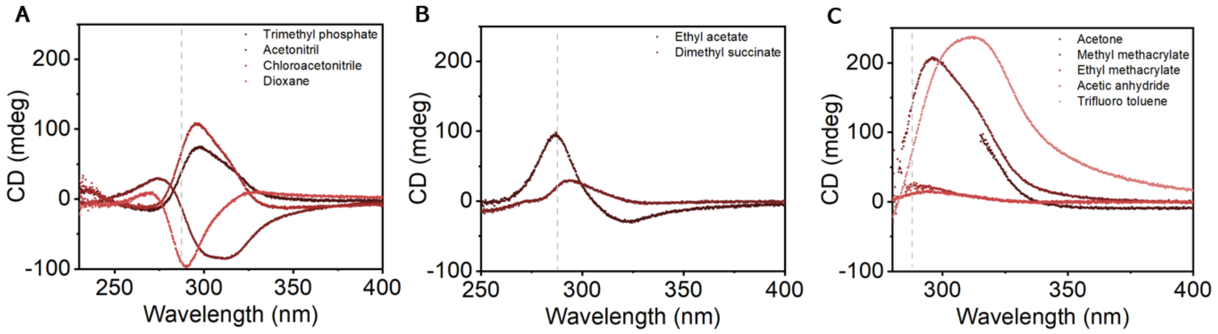

Figure S15: CD spectra of 100 µM solutions of ***S*-T** in HOA solvents grouped for solvent cut-off below (A) 230 nm, (B) 250 nm and in-between (C) 280–315 nm.

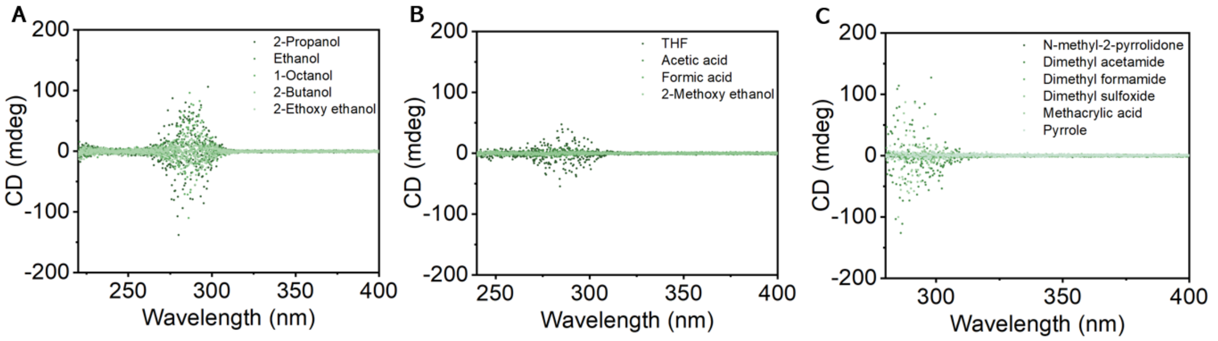

Figure S16: CD spectra of 100 µM solutions of ***S*-T** in good solvents grouped for solvent cut-off below (A) 220 nm, (B) 240 nm and (C) 280 nm.

S8.2 CD spectra of 100 µM solutions of S-A in SP, HOA and good solvents.

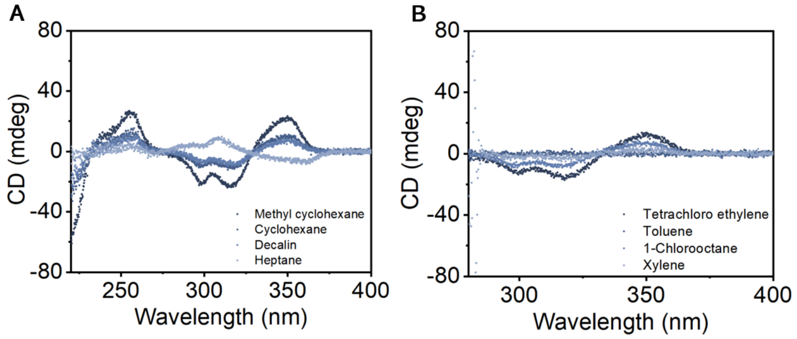

Figure S17: CD spectra of 100 µM solutions of ***S*-A** in SP solvents grouped for solvent cut-off below (A) 220 nm and (B) 250 nm.

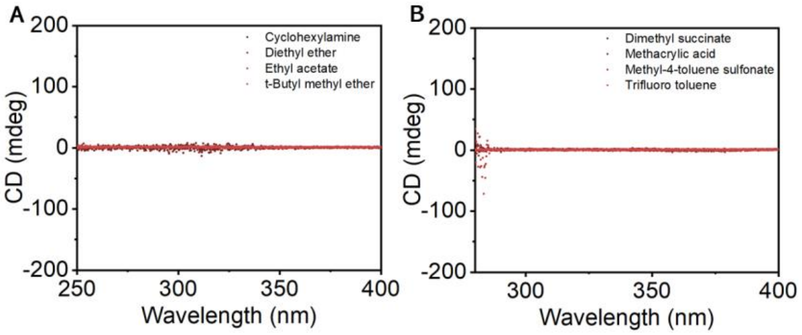

Figure S18: CD spectra of 100 µM solutions of ***S*-A** in HOA solvents grouped for solvent cut-off below (A) 250 nm and (B) 280 nm.

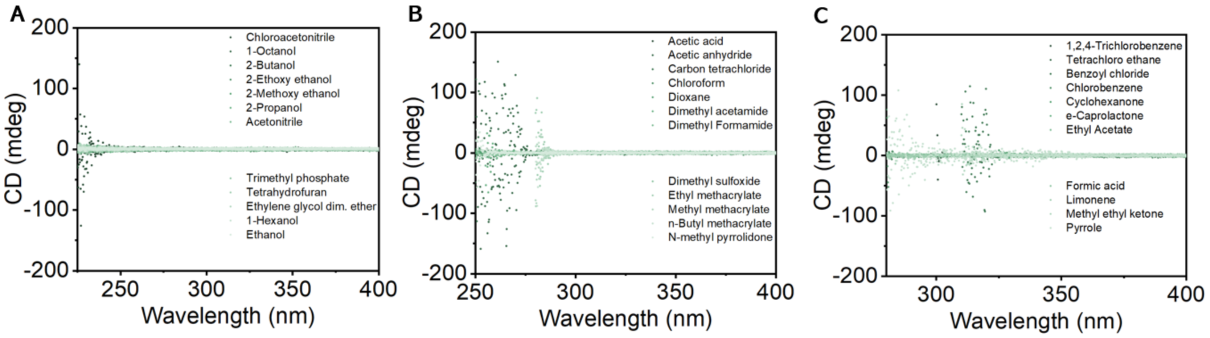

Figure S19: CD spectra of 100 µM solutions of ***S*-A** in good solvents grouped for solvent cut-off below (A) 225 nm, (B) 250–280 nm and (C) 280-315 nm.

*S8.3 CD spectra of 100 µM solutions of S-***P** *in SP, HOA and good solvents.*

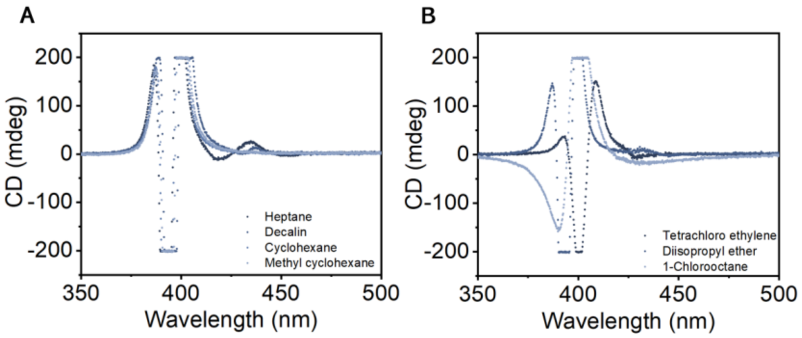

Figure S20: CD spectra of 100 µM solutions of ***S*-P** in SP solvents grouped for solvent cut-off below (A) 225 nm and (B) 250–280 nm.

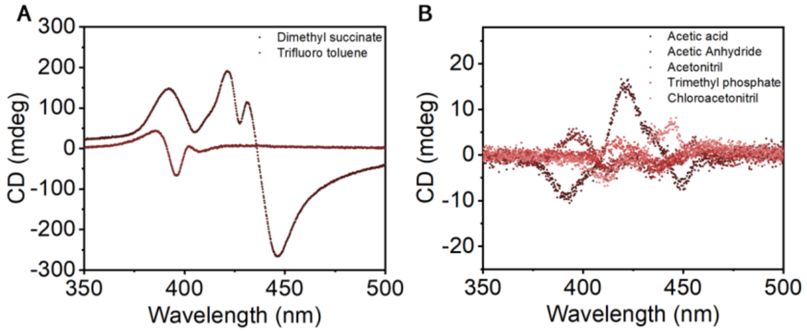

Figure S21: CD spectra of 100 µM solutions of ***S*-P** in HOA solvents grouped for (A) high and (B) low intensity CD signals.

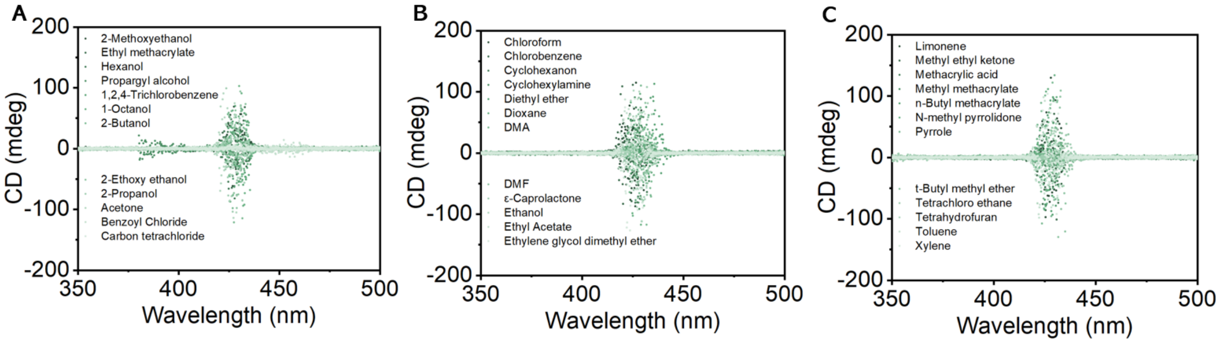

Figure S22: CD spectra of 100 µM solutions of ***S*-P** in good solvents grouped for solvent cut-off below (A) 225 nm, (B) 250–280 nm and (C) 280-315 nm.

9. References

(1) Su, H.; Jansen, S. A. H.; Schnitzer, T.; Weyandt, E.; Andreas, T. R.; Liu, J.; Vantomme, G.; Meijer, E. W. Unraveling the Complexity of Supramolecular Copolymerization Dictated by Triazine − Benzene Interactions. *J. Am. Chem. Soc* **2021**, *143*, 17128–17135. https://doi.org/10.1021/jacs.1c07690.

(2) Adelizzi, B.; Filot, I. A. W.; Palmans, A. R. A.; Meijer, E. W. Unravelling the Pathway Complexity in Conformationally Flexible N-Centered Triarylamine Trisamides. *Chem. - A Eur. J.* **2017**, *23* (25), 6103–6110. https://doi.org/10.1002/chem.201603938.

(3) Weyandt, E.; Filot, I. A. W.; Vantomme, G.; Meijer, E. W. Consequences of Amide Connectivity in the Supramolecular Polymerization of Porphyrins: Spectroscopic Observations Rationalized by Theoretical Modelling. *Chem. - A Eur. J.* **2021**, *27* (37), 9700–9707. https://doi.org/10.1002/chem.202101036.

(4) Adelizzi, B.; Aloi, A.; Van Zee, N. J.; Palmans, A. R. A.; Meijer, E. W.; Voets, I. K. Painting Supramolecular Polymers in Organic Solvents by Super-Resolution Microscopy. *ACS Nano* **2018**, *12* (5), 4431–4439. https://doi.org/10.1021/acsnano.8b00396.

(5) Weyandt, E.; Leanza, L.; Capelli, R.; Pavan, G. M.; Vantomme, G.; Meijer, E. W. Controlling the Length of Porphyrin Supramolecular Polymers via Coupled Equilibria and Dilution-Induced Supramolecular Polymerization. *Nat. Commun.* **2022**, *13* (1), 1–9. https://doi.org/10.1038/s41467-021-27831-2.

(6) Helmich, F.; Lee, C. C.; Nieuwenhuizen, M. M. L.; Gielen, J. C.; Christianen, P. C. M.; Larsen, A.; Fytas, G.; Leclère, P. E. L. G.; Schenning, A. P. H. J.; Meijer, E. W. Dilution-Induced Self-Assembly of Porphyrin Aggregates: A Consequence of Coupled Equilibria. *Angew. Chemie - Int. Ed.* **2010**, *49* (23), 3939–3942. https://doi.org/10.1002/anie.201000162.

(7) Díaz de los Ríos, M.; Hernández Ramos, E. Determination of the Hansen Solubility Parameters and the Hansen Sphere Radius with the Aid of the Solver Add-in of Microsoft Excel. *SN Appl. Sci.* **2020**, *2* (4), 1–7. https://doi.org/10.1007/s42452-020-2512-y.

(8) Louwerse, M. J.; Maldonado, A.; Rousseau, S.; Moreau-Masselon, C.; Roux, B.; Rothenberg, G. Revisiting Hansen Solubility Parameters by Including Thermodynamics. *ChemPhysChem* **2017**, *18* (21), 2999–3006. https://doi.org/10.1002/cphc.201700408.

(9) Hunter, C. A. Quantifying Intermolecular Interactions: Guidelines for the Molecular Recognition Toolbox. *Angew. Chemie - Int. Ed.* **2004**, *43* (40), 5310–5324. https://doi.org/10.1002/anie.200301739.

Appendix

A. *Hansen Solubility Parameters of solvents used in this study.*

**Solvents**	δ_D	δ_P	δ_H
1,2,4-Trichlorobenzene	20.2	4.2	3.2
1,3-Propanediol	16.8	13.5	23.2
1,4-Butanediol	16.6	11	20.9
1-Chloro-octane	16.1	5.8	1.5
1-Hexanol	15.9	5.8	12.5
1-Octanol	16	5	11.2
2-Butanol	15.8	5.7	14.5
2-Ethoxy ethanol	15.9	9.2	14.3
2-methoxy ethanol	16	9.2	16.4
2-Propanol	15.8	6.1	16.4
ε-Caprolactone	19.7	15	7.4
Acetic acid	14.5	8	13.5
Acetic anhydride	16	11.7	10.2
Acetone	15.5	10.4	7
Acetonitrile	15.3	18	6.1
Carbon tetrachloride	17.8	0	0.6
Chloroform	17.8	3.1	5.7
Chloro acetonitrile	17.4	13.6	2
Chlorobenzene	19	4.3	2
Cyclohexane	16.8	0	0.2
Cyclohexanone	17.8	8.4	5.1
Cyclohexylamine	17.2	3.1	6.5
Decalin	17.6	0	0
Diethyl Ether	14.5	2.9	4.6
Dimethyl succinate	16.1	7.7	8.8
Dioxane	17.5	1.8	9
Dimethyl acetamide	16.8	11.5	10.2
Dimethyl formamide	17.4	13.7	11.3
Dimethyl sulfoxide	18.4	16.4	10.2
Ethanol	15.8	8.8	19.4
Ethanolamine	17	15.5	21
Ethyl Acetate	15.8	5.3	7.2
Ethyl methacrylate	15.8	7.2	7.5
Ethylene glycol dimethyl ether	15.4	6.3	6
Formic acid	14.6	10	14
Heptane	15.3	0	0
Iso-Propyl Ether	15.1	3.2	3.2
Limonene	17.2	1.8	4.3
Methyl cyclochexane	16	0	1
Methyl ethyl ketone	16	9	5.1

Methacrylic acid	15.8	2.8	12
Methanol	14.7	12.3	22.3
Methyl methacrylate	15.8	6.5	5.3
Methyl-4-toluenesulfonate	19.6	15.3	3.8
n-Butyl methacrylate	15.4	5.9	5.2
N-pyrrolidon	18	12.3	7.2
Propargyl alcohol	16.1	8.7	18.8
Pyrrole	19.2	11	10
Salicylaldehyde	19	10.5	12
t-Butyl methyl ether	14.8	4.3	5
Tetrachloroethane	18.8	5.1	5.3
Tetrachloroethylene	18.3	5.7	0
Tetrahydrofuran	16.8	5.7	8
Toluene	18	1.4	2
Trifluoro toluene	17.5	8.8	0
trimethyl phosphate	15.7	10.5	10.2
Water	15.5	16	42.3
Xylene	17.6	1	3.1

B. Calculated and scaled δ_{HD} and δ_{HA} parameters of used solvents.

**Solvents**	$\boldsymbol{\delta_{HD}}$	$\boldsymbol{\delta_{HA}}$
1,2,4-Trichlorobenzene	2.6	3.9
1,3-Propanediol	12.9	41.6
1,4-Butanediol	11.6	37.5
1-Chloro-octane	1.4	1.7
1-Hexanol	7.0	22.4
1-Octanol	6.2	20.1
2-Butanol	8.1	26.0
2-Ethoxy ethanol	8.4	24.4
2-methoxy ethanol	9.6	28.0
2-Propanol	9.1	29.4
ε-Caprolactone	3.5	15.8
Acetic acid	8.2	22.3
Acetic anhydride	5.3	19.8
Acetone	3.6	13.8
Acetonitrile	3.7	10.1
Carbon tetrachloride	0.9	0.4
Chloroform	5.5	6.0
Chloro acetonitrile	1.4	2.8
Chlorobenzene	1.6	2.6
Cyclohexane	0.2	0.2
Cyclohexanone	2.6	10.0
Cyclohexylamine	3.9	10.7

Decalin	0.1	0.1
Diethyl Ether	1.9	11.2
Dimethyl succinate	4.1	18.9
Dioxane	3.7	21.8
Dimethyl acetamide	4.3	24.0
Dimethyl formamide	4.2	30.4
Dimethyl sulfoxide	4.2	24.8
Ethanol	10.8	34.8
Ethanolamine	11.4	38.7
Ethyl Acetate	3.6	14.6
Ethyl methacrylate	3.0	18.8
Ethylene glycol dimethyl ether	2.5	14.6
Formic acid	6.8	28.9
Heptane	0.4	0.3
Iso-Propyl Ether	1.3	7.8
Limonene	3.0	6.1
Methyl cyclochexane	1.2	0.9
Methyl ethyl ketone	2.6	10.0
Methacrylic acid	6.0	23.8
Methanol	12.4	40.0
Methyl methacrylate	2.3	12.5
Methyl-4-toluenesulfonate	0.6	23.3
n-Butyl methacrylate	2.1	12.8
N-pyrrolidon	3.1	16.9
Propargyl alcohol	11.5	30.6
Pyrrole	8.6	11.7
Salicylaldehyde	7.2	20.1
t-Butyl methyl ether	2.1	12.1
Tetrachloroethane	6.6	4.3
Tetrachloroethylene	0.3	0.4
Tetrahydrofuran	3.3	19.4
Toluene	1.3	3.0
Trifluoro toluene	0.2	0.7
trimethyl phosphate	3.2	32.5
Water	33.4	53.6
Xylene	2.1	4.6

C. *Solvent classification data for the concentration series of* **S-T**.

**Solvents**	δ_D	δ_P	δ_H	**25 μM**		**100 μM**		**500 μM**	
				GS	BS	GS	BS	GS	BS
1,2,4-Trichlorobenzene	20.2	4.2	3.2	1	0	0	1	0	1
1,3-Propanediol	16.8	13.5	23.2	0	0	0	0	0	0
1,4-Butanediol	16.6	11	20.9	0	0	0	0	0	0
1-Chloro-octane	16.1	5.8	1.5	0	1	0	1	0	1

1-Hexanol	15.9	5.8	12.5	1	0	1	0	1	0
1-Octanol	16	5	11.2	1	0	1	0	1	0
2-Butanol	15.8	5.7	14.5	1	0	1	0	1	0
2-Ethoxy ethanol	15.9	9.2	14.3	1	0	1	0	1	0
2-methoxy ethanol	16	9.2	16.4	1	0	1	0	0	0
2-Propanol	15.8	6.1	16.4	1	0	1	0	0	0
ε-Caprolactone	19.7	15	7.4	0*	0*	0*	0*	0*	0*
Acetic acid	14.5	8	13.5	1	0	1	0	1	0
Acetic anhydride	16	11.7	10.2	0*	0*	0*	0*	0*	0*
Acetone	15.5	10.4	7	1	0	0*	0*	0*	0*
Acetonitrile	15.3	18	6.1	0*	0*	0*	0*	0*	0*
Carbon tetrachloride	17.8	0	0.6	0	1	0	1	0	1
Chloroform	17.8	3.1	5.7	1	0	0	1	0	1
Chloro acetonitrile	17.4	13.6	2	0*	0*	0*	0*	0*	0*
Chlorobenzene	19	4.3	2	1	0	0	1	0	1
Cyclohexane	16.8	0	0.2	0	1	0	1	0	1
Cyclohexanone	17.8	8.4	5.1	1	0	0	0	0	0
Cyclohexylamine	17.2	3.1	6.5	0	0	0	0	0	0
Decalin	17.6	0	0	0	1	0	1	0	1
Diethyl Ether	14.5	2.9	4.6	0	1	0	1	0	1
Dimethyl succinate	16.1	7.7	8.8	0*	0*	0*	0*	0*	0*
Dioxane	17.5	1.8	9	1	0	0*	0*	0*	0*
Dimethyl acetamide	16.8	11.5	10.2	1	0	1	0	1	0
Dimethyl formamide	17.4	13.7	11.3	1	0	1	0	1	0
Dimethyl sulfoxide	18.4	16.4	10.2	1	0	1	0	0	0
Ethanol	15.8	8.8	19.4	1	0	0	0	0	0
Ethanolamine	17	15.5	21	0	0	0	0	0	0
Ethyl Acetate	15.8	5.3	7.2	0*	0*	0*	0*	0*	0*
Ethyl methacrylate	15.8	7.2	7.5	1	0	0*	0*	0*	0*
Ethylene glycol dimethyl ether	15.4	6.3	6	1	0	0	0	0	0
Formic acid	14.6	10	14	1	0	1	0	1	0
Heptane	15.3	0	0	0	1	0	1	0	1
Iso-Propyl Ether	15.1	3.2	3.2	0	1	0	1	0	1
Limonene	17.2	1.8	4.3	0	1	0	1	0	1
Methyl cyclochexane	16	0	1	0	1	0	1	0	1
Methyl ethyl ketone	16	9	5.1	1	0	0	0	0*	0*
Methacrylic acid	15.8	2.8	12	0	0	0	0	0	0
Methanol	14.7	12.3	22.3	0	0	0	0	0	0
Methyl methacrylate	15.8	6.5	5.3	0*	0*	0*	0*	0*	0*
Methyl-4-toluenesulfonate	19.6	15.3	3.8	0*	0*	0*	0*	0*	0*
n-Butyl methacrylate	15.4	5.9	5.2	1	0	0	1	0	1
N-pyrrolidon	18	12.3	7.2	1	0	1	0	1	0
Propargyl alcohol	16.1	8.7	18.8	1	0	0	0	0	0
Pyrrole	19.2	11	10	1	0	1	0	1	0

Salicylaldehyde	19	10.5	12	1	0	1	0	0	0
t-Butyl methyl ether	14.8	4.3	5	0	1	0	1	0	1
Tetrachloroethane	18.8	5.1	5.3	1	0	1	0	0	1
Tetrachloroethylene	18.3	5.7	0	0	1	0	1	0	1
Tetrahydrofuran	16.8	5.7	8	1	0	1	0	1	0
Toluene	18	1.4	2	0	1	0	1	0	1
Trifluoro toluene	17.5	8.8	0	0*	0*	0*	0*	0*	0*
trimethyl phosphate	15.7	10.5	10.2	0*	0*	0*	0*	0*	0*
Water	15.5	16	42.3	0	0	0	0	0	0
Xylene	17.6	1	3.1	0	1	0	1	0	1

*In these solvents fibrous precipitates or HOAs were observed and are classified as HOA solvents. The remaining solvents with an assignation of “0” for both the green sphere as blue sphere are classified as bad solvents.

*D. Solvent classification data for the temperature series **S-T**.*

**Solvents**	δ_D	δ_P	δ_H	**20 °C**		**90 °C**	
				GS	BS	GS	BS
1,2,4-Trichlorobenzene	20.2	4.2	3.2	0	1	1	0
1,3-Propanediol	16.8	13.5	23.2	0	0	0	0
1,4-Butanediol	16.6	11	20.9	0	0	0	0
1-Chloro-octane	16.1	5.8	1.5	0	1	0	1
1-Hexanol	15.9	5.8	12.5	1	0	1	0
1-Octanol	16	5	11.2	1	0	1	0
2-Butanol	15.8	5.7	14.5	1	0	1	0
2-Ethoxy ethanol	15.9	9.2	14.3	1	0	1	0
2-methoxy ethanol	16	9.2	16.4	1	0	1	0
2-Propanol	15.8	6.1	16.4	1	0	1	0
ε-Caprolactone	19.7	15	7.4	0*	0*	0	0
Acetic acid	14.5	8	13.5	1	0	1	0
Acetic anhydride	16	11.7	10.2	0*	0*	0	0
Acetone	15.5	10.4	7	0*	0*	1	0
Acetonitrile	15.3	18	6.1	0*	0*	1	0
Carbon tetrachloride	17.8	0	0.6	0	1	0	1
Chloroform	17.8	3.1	5.7	0	1	1	0
Chloro acetonitrile	17.4	13.6	2	0*	0*	0	0
Chlorobenzene	19	4.3	2	0	1	1	0
Cyclohexane	16.8	0	0.2	0	1	0	1
Cyclohexanone	17.8	8.4	5.1	0	0	1	0
Cyclohexylamine	17.2	3.1	6.5	0	0	1	0
Decalin	17.6	0	0	0	1	0	1
Diethyl Ether	14.5	2.9	4.6	0	1	0	1
Dimethyl succinate	16.1	7.7	8.8	0*	0*	1	0
Dioxane	17.5	1.8	9	0*	0*	1	0
Dimethyl acetamide	16.8	11.5	10.2	1	0	1	0

Dimethyl formamide	17.4	13.7	11.3	1	0	1	0
Dimethyl sulfoxide	18.4	16.4	10.2	1	0	1	0
Ethanol	15.8	8.8	19.4	0	0	1	0
Ethanolamine	17	15.5	21	0	0	0	0
Ethyl Acetate	15.8	5.3	7.2	0*	0*	1	0
Ethyl methacrylate	15.8	7.2	7.5	0*	0*	1	0
Ethylene glycol dimethyl ether	15.4	6.3	6	0	0	1	0
Formic acid	14.6	10	14	1	0	1	0
Heptane	15.3	0	0	0	1	0	1
Iso-Propyl Ether	15.1	3.2	3.2	0	1	0	1
Limonene	17.2	1.8	4.3	0	1	0	1
Methyl cyclohexane	16	0	1	0	1	0	1
Methyl ethyl ketone	16	9	5.1	0	0	1	0
Methacrylic acid	15.8	2.8	12	0	0	0	0
Methanol	14.7	12.3	22.3	0	0	0	0
Methyl methacrylate	15.8	6.5	5.3	0*	0*	1	0
Methyl-4-toluenesulfonate	19.6	15.3	3.8	0*	0*	0	0
n-Butyl methacrylate	15.4	5.9	5.2	0	1	1	0
N-pyrrolidon	18	12.3	7.2	1	0	1	0
Propargyl alcohol	16.1	8.7	18.8	0	0	1	0
Pyrrole	19.2	11	10	1	0	1	0
Salicylaldehyde	19	10.5	12	1	0	1	0
t-Butyl methyl ether	14.8	4.3	5	0	1	0	1
Tetrachloroethane	18.8	5.1	5.3	1	0	1	0
Tetrachloroethylene	18.3	5.7	0	0	1	0	1
Tetrahydrofuran	16.8	5.7	8	1	0	1	0
Toluene	18	1.4	2	0	1	0	1
Trifluoro toluene	17.5	8.8	0	0*	0*	0	0
trimethyl phosphate	15.7	10.5	10.2	0*	0*	0	0
Water	15.5	16	42.3	0	0	0	0
Xylene	17.6	1	3.1	0	1	0	1

*In these solvents fibrous precipitates or HOAs were observed and are classified as HOA solvents. The remaining solvents with an assignation of "0" for both the green sphere as blue sphere are classified as bad solvents.

*E. Solvent classification data for 100 µM solutions of **S-P** and **S-A**.*

**Solvents**	δ_D	δ_P	δ_H	**S-P**		**S-A**	
				GS	BS	GS	BS
1,2,4-Trichlorobenzene	20.2	4.2	3.2	1	0	1	0
1,3-Propanediol	16.8	13.5	23.2	0	0	0	0
1,4-Butanediol	16.6	11	20.9	0	0	0	0
1-Chloro-octane	16.1	5.8	1.5	0	1	0	1
1-Hexanol	15.9	5.8	12.5	1	0	1	0
1-Octanol	16	5	11.2	1	0	1	0
2-Butanol	15.8	5.7	14.5	1	0	1	0

2-Ethoxy ethanol	15.9	9.2	14.3	1	0	1	0
2-methoxy ethanol	16	9.2	16.4	0	0	1	0
2-Propanol	15.8	6.1	16.4	1	0	1	0
ε-Caprolactone	19.7	15	7.4	1	0	0	0
Acetic acid	14.5	8	13.5	0*	0*	1	0
Acetic anhydride	16	11.7	10.2	0*	0*	1	0
Acetone	15.5	10.4	7	1	0	1	0
Acetonitrile	15.3	18	6.1	0*	0*	1	0
Carbon tetrachloride	17.8	0	0.6	0	0	0	0
Chloroform	17.8	3.1	5.7	1	0	1	0
Chloro acetonitrile	17.4	13.6	2	0*	0*	0	0
Chlorobenzene	19	4.3	2	1	0	1	0
Cyclohexane	16.8	0	0.2	0	1	0	1
Cyclohexanone	17.8	8.4	5.1	1	0	1	0
Cyclohexylamine	17.2	3.1	6.5	1	0	0*	0*
Decalin	17.6	0	0	0	1	0	1
Diethyl Ether	14.5	2.9	4.6	1	0	0*	0*
Dimethyl succinate	16.1	7.7	8.8	0*	0*	0*	0*
Dioxane	17.5	1.8	9	1	0	0	0
Dimethyl acetamide	16.8	11.5	10.2	1	0	1	0
Dimethyl formamide	17.4	13.7	11.3	1	0	1	0
Dimethyl sulfoxide	18.4	16.4	10.2	0	0	1	0
Ethanol	15.8	8.8	19.4	1	0	0	0
Ethanolamine	17	15.5	21	0	0	0	0
Ethyl Acetate	15.8	5.3	7.2	1	0	0*	0*
Ethyl methacrylate	15.8	7.2	7.5	1	0	1	0
Ethylene glycol dimethyl ether	15.4	6.3	6	1	0	1	0
Formic acid	14.6	10	14	0	0	1	0
Heptane	15.3	0	0	0	1	0	1
Iso-Propyl Ether	15.1	3.2	3.2	0	1	0	0
Limonene	17.2	1.8	4.3	1	0	1	0
Methyl cyclochexane	16	0	1	0	1	0	1
Methyl ethyl ketone	16	9	5.1	1	0	1	0
Methacrylic acid	15.8	2.8	12	1	0	0*	0*
Methanol	14.7	12.3	22.3	0	0	0	0
Methyl methacrylate	15.8	6.5	5.3	1	0	1	0
Methyl-4-toluenesulfonate	19.6	15.3	3.8	0	0	0*	0*
n-Butyl methacrylate	15.4	5.9	5.2	1	0	1	0
N-pyrrolidon	18	12.3	7.2	1	0	1	0
Propargyl alcohol	16.1	8.7	18.8	0	0	0	0
Pyrrole	19.2	11	10	1	0	1	0
Salicylaldehyde	19	10.5	12	0	0	1	0
t-Butyl methyl ether	14.8	4.3	5	1	0	0*	0*
Tetrachloroethane	18.8	5.1	5.3	1	0	1	0

Tetrachloroethylene	18.3	5.7	0	0	1	0	1
Tetrahydrofuran	16.8	5.7	8	1	0	1	0
Toluene	18	1.4	2	1	0	0	1
Trifluoro toluene	17.5	8.8	0	0*	0*	0*	0*
trimethyl phosphate	15.7	10.5	10.2	0*	0*	1	0
Water	15.5	16	42.3	0	0	0	0
Xylene	17.6	1	3.1	1	0	0	1

*In these solvents fibrous precipitates or HOAs were observed and are classified as HOA solvents. The remaining solvents with an assignation of “0” for both the green sphere as blue sphere are classified as bad solvents.
